# Modulation of blood inflammatory markers by benralizumab in patients with eosinophilic airway diseases

**DOI:** 10.1186/s12931-018-0968-8

**Published:** 2019-01-18

**Authors:** Sriram Sridhar, Hao Liu, Tuyet-Hang Pham, Gautam Damera, Paul Newbold

**Affiliations:** MedImmune LLC, One MedImmune Way, #4552B, Gaithersburg, MD USA

**Keywords:** Asthma, Basophils, Benralizumab, Blood, Chronic obstructive pulmonary disease, Eosinophils, Gene expression, Gene set variation analysis, Inflammatory markers, Proteomics

## Abstract

**Background:**

Benralizumab, a humanized, afucosylated, monoclonal antibody that targets interleukin-5 receptor α, depletes eosinophils and basophils by enhanced antibody-dependent cell-mediated cytotoxicity. It demonstrated efficacy for patients with moderate to severe asthma and, in a Phase IIa trial, for chronic obstructive pulmonary disease (COPD) with eosinophilic inflammation. We investigated effects of benralizumab 100 mg every 8 weeks (first three doses every 4 weeks) subcutaneous on blood inflammatory markers through proteomic and gene-expression analyses collected during two Phase II studies of patients with eosinophilic asthma and eosinophilic COPD.

**Methods:**

Serum samples for proteomic analysis and whole blood for gene expression analysis were collected at baseline and 52 weeks (asthma study) or 32 weeks (COPD study) post-treatment. Proteomic analyses were conducted on a custom set of 90 and 147 Rules-Based Medicine analytes for asthma and COPD, respectively. Gene expression was profiled by Affymetrix Human Genome U133 plus 2 arrays (~ 54 K probes). Gene set variation analysis (GSVA) was used to determine transcriptomic activity of immune signatures. Treatment-related differences between analytes, genes, and gene signatures were analyzed for the overall population and for patient subgroups stratified by baseline blood eosinophil count (eosinophil-high [≥300 cells/μL] and eosinophil-low [< 300 cells/μL]) via t-test and repeated measures analysis of variance.

**Results:**

Eosinophil chemokines eotaxin-1 and eotaxin-2 were significantly upregulated (false discovery rate [FDR] < 0.05) by approximately 2.1- and 1.4-fold in the asthma study and by 2.3- and 1.7-fold in the COPD study following benralizumab treatment. Magnitude of upregulation of these two chemokines was greater for eosinophil-high patients than eosinophil-low patients in both studies. Benralizumab was associated with significant reductions (FDR < 0.05) in expression of genes associated with eosinophils and basophils, such as CLC, IL-5Rα, and PRSS33; immune-signaling complex genes (FCER1A); G-protein–coupled receptor genes (HRH4, ADORA3, P2RY14); and further immune-related genes (ALOX15 and OLIG2). The magnitude of downregulation of gene expression was greater for eosinophil-high than eosinophil-low patients. GSVA on immune signatures indicated significant treatment reductions (FDR < 0.05) in eosinophil-associated signatures.

**Conclusions:**

Benralizumab is highly selective, modulating blood proteins or genes associated with eosinophils or basophils. Modulated protein and gene expression patterns are most prominently altered in eosinophil-high vs. eosinophil-low patients.

**Trial registration:**

NCT01227278 and NCT01238861.

**Electronic supplementary material:**

The online version of this article (10.1186/s12931-018-0968-8) contains supplementary material, which is available to authorized users.

## Background

Asthma and chronic obstructive pulmonary disease (COPD), two of the most common chronic respiratory diseases, are a significant cause of morbidity and mortality worldwide [1]. Patients with inadequately controlled symptoms of asthma and COPD are at a particularly high risk of exacerbations, hospitalization and mortality and often have substantially impaired health-related quality of life [2, 3]. Consequently, the economic burden and health care costs associated with both conditions are considerable, with total annual costs in the United States alone amounting to $81.9 billion and $52 billion for asthma and COPD, respectively [4, 5].

Subgrouping of asthma and COPD by phenotype can help deliver tailored treatments and guide the selection of optimal therapeutic agents, thereby lowering disease burden and health care costs [6, 7]. Indeed, a wealth of evidence demonstrates that an eosinophilic phenotype exists in a significant percentage of patients with both asthma and COPD. In asthma, persistent eosinophilic airway inflammation is present in up to 50% of patients [8], and is associated with increased severity, exacerbations, decreased lung function, and mortality [9–12]. Likewise, eosinophilic inflammation has been observed in 20–40% of patients with COPD [13]. Notably, while eosinophilic COPD has frequently been labeled as part of the asthma–COPD overlap, it now appears to be a distinct patient subgroup with an increased corticosteroid response [13]. Indeed, a management strategy of increasing therapy with corticosteroids used to control sputum eosinophilia greater than 3% in patients with COPD resulted in a reduction in the frequency of COPD exacerbations requiring hospital admission [14]. However, while corticosteroid treatment generally results in a reduction in eosinophils for both patients with asthma and COPD, approximately 50% of severe asthmatics experience exacerbations and symptoms with the presence of persistent eosinophils despite receiving high-dosage inhaled corticosteroids (ICS) [15–17], and approximately 30–40% of patients with COPD continue to have moderate or severe exacerbations despite receiving triple inhaled therapy [18]. Furthermore, long-term systemic corticosteroid therapy is associated with substantial adverse effects, including hypothalamic-pituitary-adrenal axis suppression and osteoporosis [19, 20]. Therefore, there remains a need for treatment strategies that reduce eosinophilic inflammation without corticosteroid-induced adverse effects.

Benralizumab is a humanized, afucosylated, monoclonal antibody that binds to interleukin-5 receptor α (anti–IL-5Rα) and induces direct, rapid, and nearly complete depletion of blood eosinophils via enhanced antibody-dependent cell-mediated cytotoxicity, an apoptotic process of eosinophil elimination involving immune effector cells, such as natural killer cells and macrophages [21, 22]. In the United States, benralizumab is approved for add-on maintenance treatment of patients with severe asthma aged 12 years and older and with an eosinophilic phenotype [23].

Results from a Phase IIb dose-ranging study of patients with severe uncontrolled asthma (NCT01238861) demonstrated that benralizumab, dosed at 20 mg or 100 mg subcutaneously, and administered every 4 weeks for the first three doses and then every 8 weeks thereafter (hereafter defined as Q8W) for 1 year, significantly reduced asthma exacerbations in adults with uncontrolled asthma with baseline blood eosinophils of at least 300 cells/μL [24]. However, results from a Phase IIa trial of patients with eosinophilic COPD (defined as patients with a sputum eosinophil count of at least 3% within the previous year prior to randomization; NCT01227278) reported that benralizumab 100 mg Q8W for 1 year did not reduce the annualized rate of acute exacerbations. Nonetheless, a numerical, albeit, non-significant improvement in acute exacerbations of COPD was reported for benralizumab-treated patients with baseline blood eosinophil concentrations greater than 200 cells/μL [25]. More recently, the two Phase III studies evaluating benralizumab for patients with moderate to very severe COPD did not meet the primary endpoint of a statistically significant reduction of exacerbations [26, 27].

Phase II studies in asthma and COPD established that benralizumab induces depletion of blood eosinophils and basophils [24, 25, 28]. In the severe asthma Phase IIb study, treatment with both benralizumab 20 and 100 mg Q8W resulted in nearly complete depletion of blood eosinophils, which was sustained throughout the treatment period [24]. Results of an exploratory analysis of the same study demonstrated that blood basophils (measured using flow cytometry) were also reduced at the end of treatment [28]. Similarly, in the COPD Phase IIa study, benralizumab 100 mg Q8W resulted in nearly complete depletion of blood eosinophils, which was maintained throughout the treatment period [25].

The objective of our study was to gain further insight into the mechanism of action of benralizumab. Hence, we assessed whether there are additional effects of benralizumab on inflammatory signals or on the inflammatory cell profile, besides its reported effect of eosinophil and basophil depletion. We therefore investigated the effects of benralizumab 100 mg Q8W vs. placebo on blood inflammatory markers by proteomic and gene array analyses in samples collected during the Phase II studies in asthma and COPD.

## Methods

### Study design and patients

Full details of the study designs have been published [24, 25]. Both were Phase II, randomized, double-blind, placebo-controlled, multicenter studies. The asthma study was a dose-ranging study that evaluated the efficacy and safety of multiple-dose subcutaneous (SC) administration of benralizumab (2, 20, or 100 mg) Q8W in adult patients with uncontrolled asthma [24]. The COPD study investigated the efficacy of multiple SC doses of benralizumab 100 mg Q8W in adult patients with moderate to severe COPD [25].

In the asthma study, eligible participants were adults aged 18–75 years with uncontrolled asthma using medium- or high-dosage ICS and long-acting β_2_-agonists and who had experienced two to six exacerbations in the past year. Patients were stratified by baseline fraction of exhaled nitric oxide (FeNO < 50 ppb or ≥ 50 ppb) or eosinophilic status based on the eosinophil/lymphocyte and eosinophil/neutrophil combined ratio (ELEN) index (an algorithm to predict elevated sputum eosinophils; sputum eosinophils < 2% [eosinophilic-negative] or sputum eosinophils ≥2% [eosinophilic-positive]) [29]. The ELEN index was used to predict elevated sputum eosinophils to identify severe asthma patients with an eosinophilic phenotype, as routine collection of induced sputum from patients attending clinics is not always feasible. Eosinophilic-positive patients were randomized in a 1:1:1:1 ratio to receive SC benralizumab (2, 20, or 100 mg) or placebo; eosinophilic-negative patients were randomized in a 1:1 ratio to receive SC benralizumab 100 mg or placebo. Benralizumab was administered Q4W for the first 3 doses on Weeks 1, 4, and 8 and then Q8W thereafter at Weeks 16, 24, 32, and 40 (Additional file 1: Figure S1).

In the COPD study, eligible participants were adults aged 40–85 years, with moderate to severe COPD, at least one acute exacerbation of COPD, and a sputum eosinophil count ≥3.0% within the previous year or at screening. Patients were randomized in a 1:1 ratio to receive either benralizumab 100 mg or placebo Q4W for the first 3 doses at Weeks 1, 4, and 8 and then Q8W thereafter at Weeks 16, 24, 32, 40, and 48 (Additional file 1: Figure S1).

### Blood sample collection

For proteomic and gene expression analyses, venous blood samples were collected from patients in both studies prior to dose administration at baseline and at each subsequent visit.

For proteomic analyses, whole blood was collected from patients in serum separator tubes, allowed to clot for 30 min, and then centrifuged within an hour of collection. Serum aliquots were stored at − 20 °C at the clinical site until they were shipped to the central laboratory (Eurofins, Luxembourg) on dry ice. Once received, the aliquots were stored at − 80 °C. After study completion, the serum aliquots were shipped to MedImmune (Mountain View, CA) for long term storage at − 80 °C until biomarker assessment.

For gene expression analyses, whole blood was collected in 2.5 mL PAXgene Blood RNA tubes for RNA transcript profiling. RNA was isolated and processed to be run on Affymetrix Human Genome U133 plus 2.0 microarrays by Assuragen, Inc. (Austin, TX).

For proteomic and gene array analyses, only patient samples collected at screening in both studies and at end of treatment (Week 52 in the asthma study and Week 32 in the COPD study) were considered.

### Proteomic analysis

A custom set of protein analytes (Rules-Based Medicine [RBM], Austin, TX) were measured in serum samples collected from patients in the asthma (90 analytes) and COPD (147 analytes) cohorts. RBM serum protein data was generated on a Luminex-based platform and assayed using proprietary multiplex assay reagents. The list of RBM serum proteins that were analyzed for the asthma and COPD studies and demonstrated a change from their baseline values is provided in Additional file 2: Table S1.

### Gene expression and gene signature analysis

Total RNA was extracted from cells in whole blood samples collected into PAXgene tubes from patients. RNA quality was confirmed using the RNA integrity number (RIN > 6) generated from the Agilent 2100 Bioanalyzer with the RNA 6000 Nano LabChip (Agilent Technologies, Santa Clara, CA). Gene expression was assessed on Affymetrix Human Genome U133 plus 2.0 microarrays (approximately 54 K probe sets mapping to approximately 20 K unique genes). Gene set variation analysis (GSVA) [30] was used to determine the modulation of immune-related signatures of interest in benralizumab-treated patients compared with placebo-treated patients in both the asthma and COPD cohorts. Immune-related signatures included a collection of signatures that were curated from both publicly available literature and an internally compiled set of genes from cell stimulation experiments. These compilations comprised genes identified as markers of specific immune cell types. Included in these gene signatures were two internally defined gene eosinophil signatures (eosinophil gene signature #1 and 2-gene eosinophil signature) along with two from previously published work (eosinophil gene signature #2 and eosinophil gene signature #3) [31, 32]. GSVA scores were calculated for internally defined or previously published gene signatures for all patients. Signature scores ranged from − 1 to 1, with negative scores indicating relative decreases in signature expression and positive scores indicating relative elevations in signature expression.

### Statistical analyses

Changes in protein analyte concentrations were determined through comparison of post-treatment (52 weeks in the asthma study and 32 weeks in the COPD study) analyte concentrations with baseline concentrations. The mean change in analyte concentration post-treatment was assessed between treatment arms across all patients and within the subset of patients with high blood eosinophil count (eosinophil-high: eosinophil count ≥300 cells/μL) and low blood eosinophil count (eosinophil-low: eosinophil count < 300 cells/μL) via t-test. False discovery rates (FDRs) were determined using the Benjamini-Hochberg procedure to account for multiple comparisons. Analyte changes with an FDR < 0.05 were considered statistically significant. For analytes below the lower limit of quantification (LLOQ), analyte values were set to 50% of the LLOQ value.

Changes in gene expression and the GSVA scores of immune cell gene signature were determined through comparison of post-treatment expression (52 weeks in the asthma study and 32 weeks in the COPD study) with baseline expression using a repeated-measures analysis of variance (ANOVA), which assessed significant treatment-specific changes in gene or signature expression and accounted for patients being sampled at multiple timepoints in the two treatment arms. Gene expression data were processed using frozen robust multiarray analysis (fRMA). When multiple probe sets were mapped to the same gene, the probe set with the highest interquartile range was selected as representative for the gene. This resulted in a gene expression dataset consisting of unique gene symbols and their expression across all samples. Genes were considered to indicate a statistically significant difference in the treatment group if they changed by at least 1.5-fold in the benralizumab-treated arm post-treatment compared with baseline, with an FDR < 0.05 based on ANOVA. FDRs for gene signature differences were determined using the Benjamini-Hochberg procedure. In addition, changes in the benralizumab-treated group were measured in patient subgroups with high and low eosinophil counts. However, since subgroup sample sizes in the COPD study were small, the study was not adequately powered to determine differences between blood eosinophil-high and eosinophil-low subgroups based on FDR. As such, the significance of expression changes in eosinophil-high and eosinophil-low subgroups was determined using a raw ANOVA *p-*value < 0.05.

In addition, we conducted *post-hoc* analyses to determine whether blood eosinophil counts affect the eosinophil gene signature. After adjusting for baseline blood eosinophil counts in the repeated measures ANOVA, we analyzed the effect of benralizumab treatment (vs. placebo) on the internal eosinophil signature score in both the Phase II asthma and COPD cohorts. The ANOVA *p*-value calculated was adjusted for multiple comparisons.

## Results

### Patient disposition

Of the 609 patients who were included in the asthma study, 444 patients received either benralizumab 100 mg SC or placebo (222 in each treatment arm) [24]. Of these 444 patients, blood samples for proteomic and gene expression analyses were available for 395 patients and 326 patients, respectively. Of the 101 patients included in the COPD study [25], blood samples for proteomic and gene expression analyses were available for 84 patients and 78 patients, respectively (Table 1).

**Table 1:** Patient disposition for proteomic and gene expression analyses

	Asthma (protein)	Asthma (gene expression)	COPD (protein)	COPD (gene expression)
Total patients	395	326	84	78
Placebo (all)	195	163	41^b^	40^b^
Placebo (EOS-high)	75	60	13	11
Placebo (EOS-low)	121	103	24	25
Benralizumab (all)	200^a^	163^a^	43^b^	38^b^
Benralizumab (EOS-high)	90	69	15	16
Benralizumab (EOS-low)	110	93	22	18

EOS-high: eosinophil count ≥300 cells/μL

EOS-low: eosinophil count < 300 cells/μL

*COPD* chronic obstructive pulmonary disease, *EOS* eosinophils

^a^One patient with asthma did not have eosinophil status and could not be designated into a subgroup for proteomic or gene expression analysis

^b^10 patients with COPD did not have eosinophil status and could not be designated into subgroups for proteomic analysis; eight patients could not be designated into subgroups for gene expression analysis

### Serum protein markers of response

**Fig. 1:**
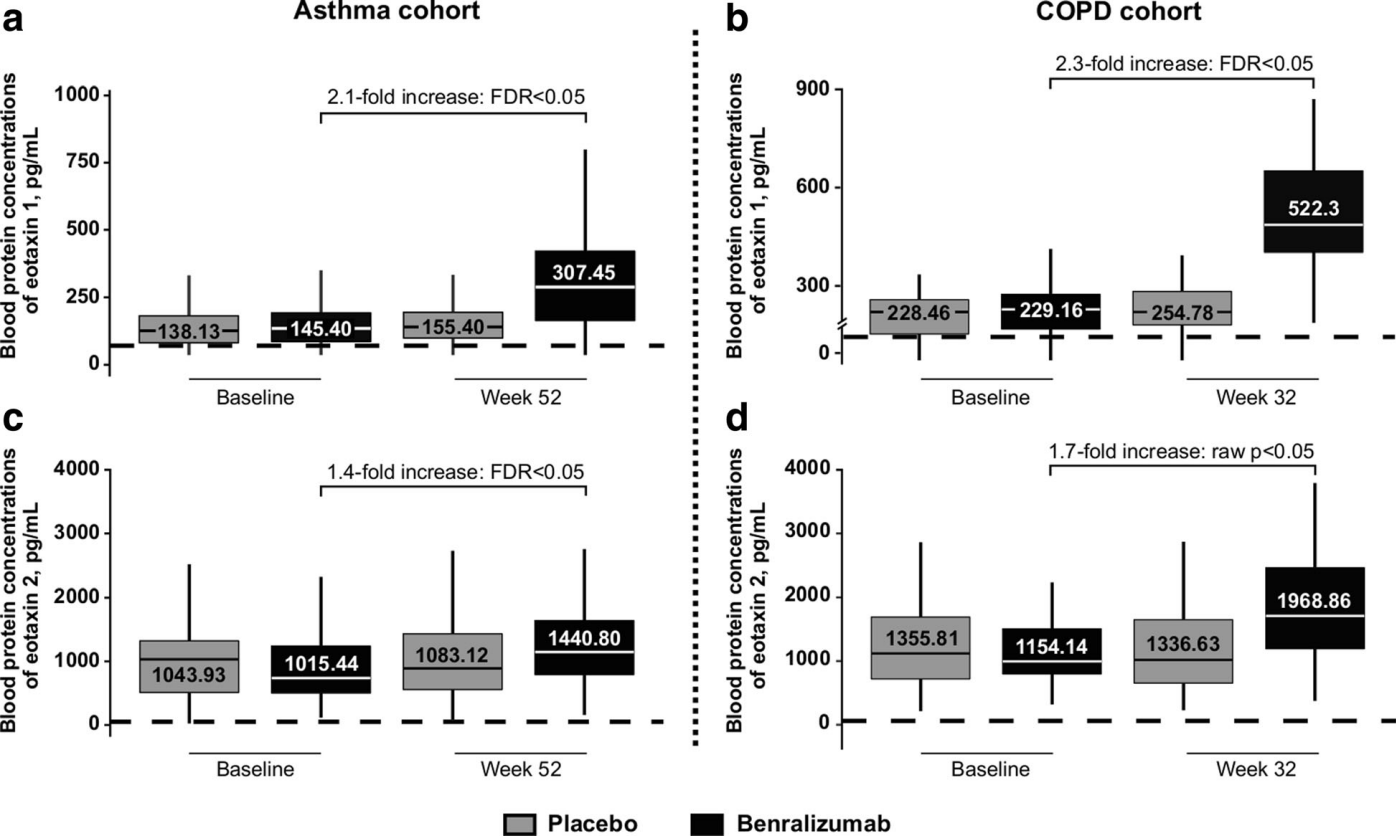
Protein analyte concentrations of eotaxin-1 and eotaxin-2 in patients with asthma and COPD. Concentrations of eotaxin-1 and eotaxin-2 at baseline and after 52 weeks of treatment with benralizumab vs. placebo in the overall population of the asthma cohort (**a** and **c**). Concentrations of eotaxin-1 and eotaxin-2 at baseline and after 32 weeks of treatment with benralizumab vs. placebo in the overall population of the COPD cohort (**b** and **d**). Boxplots display the 25th–75th percentile values, with bars denoting median values. Boxes are labeled with the mean concentration per treatment arm. The dotted line denotes the analyte LLOQ. COPD, chronic obstructive pulmonary disease; FDR, false discovery rate; LLOQ, lower limit of quantification

Of the 90 and 147 protein analytes assessed for the asthma cohort and COPD cohort, respectively, only eotaxin-1 was significantly upregulated (> 1.5 absolute–fold change following benralizumab treatment compared with placebo in both patient cohorts; FDR < 0.05; Fig. 1a, b). Eotaxin-2 was also significantly upregulated in the asthma cohort (FDR < 0.05) and the COPD cohort (raw *p* < 0.05, but not FDR) following benralizumab treatment (Fig. 1c, d). The magnitude of upregulation of eotaxin-1 was greater than that of eotaxin-2; blood concentrations of eotaxin-1 and eotaxin-2 were elevated 2.1- and 1.4-fold after 52 weeks of treatment with benralizumab in the asthma cohort and 2.3- and 1.7-fold after 32 weeks of treatment with benralizumab in the COPD cohort. Eotaxin-1 and eotaxin-2 concentrations did not significantly change in the placebo group of either cohort (Fig. 1). Overall, the magnitude of upregulation of eotaxin-1 and eotaxin-2 was greater in the eosinophil-high group than the eosinophil-low group in both asthma and COPD patients (Additional file 3: Figure S2).

Brain-derived neurotrophic factor (BDNF) was significantly downregulated in the asthma cohort following benralizumab treatment (FDR < 0.05), although the magnitude of change was modest (10% decrease post-treatment) (Additional file 4: Figure S3). No other analytes were significantly altered in either the benralizumab or placebo treatment groups (Additional file 2: Table S1).

### Gene expression markers of response

A significant downregulation in the expression of several genes was observed for patients treated with benralizumab, all of which were associated with either eosinophil or basophil biology (Table 2). In general, the magnitude of downregulation of gene expression in the eosinophil-high group was greater than that in the eosinophil-low group following treatment with benralizumab for patients with both asthma and COPD (data not shown).

**Table 2:** Fold change from baseline in gene expression in patients with asthma and COPD

Genes	Asthma cohort		COPD cohort		Gene association with eosinophils/basophils
	Benralizumab	Placebo	Benralizumab	Placebo	
*CLC* ^a^	−4.68	−1.08	−5.32	−1.02	Eosinophil or basophil marker [46–49]
*PRSS33* ^a^	−2.45	1.04	−2.74	−1.1	Eosinophil or basophil marker [50]
*OLIG2* ^a^	−2.01	−1.05	−1.91	−1.01	Expressed in eosinophils and associated with the control of SIGLEC 8 expression [51]
*FCER1A* ^b^	−1.96	−1.11	−1.84	−1.18	Fc epsilon receptor 1A, the high affinity receptor for IgE and expressed on eosinophils and basophils [52]
*HRH4* ^b^	−1.91	−1.03	−1.94	−1.17	Histamine receptor 4 expressed on eosinophils [53]
*CCR3* ^b^	−1.86	−1.14	−1.85	1.02	Chemokine receptor for eosinophils and basophils [54, 55]
*IL5Rα* ^a^	−1.82	−1.06	−1.97	1.03	IL-5 receptor alpha expressed on eosinophils and basophils [56–58]
*IDO1* ^b^	−1.75	1.03	−1.83	−1.00	Eosinophil or basophil marker [59]
*P2RY14* ^c^	−1.71	−1.12	−1.70	−1.20	A purine receptor, P2Y14. Upregulated in eosinophils after allergen challenge and an asthma risk gene [60]
*OLIG1* ^b^	−1.70	1.04	−2.22	−1.28	Eosinophil marker [61]
*ADORA3* ^a^	−1.60	−1.02	−1.64	1.03	Adenosine A3 receptor expressed on eosinophils [62, 63]
*CD9* ^b^	−1.58	−1.21	−1.47	1.02	Motility and signal transduction expressed on eosinophils, basophils, and platelets [64]
*ALOX15* ^b^	−1.57	1	−1.64	−1.02	Gene for 15-lipoxygenase, an enzyme abundantly expressed in eosinophil [65]
*CD24* ^b^	−1.54	−1.06	−1.72	−1.11	Sialoglycoprotein expressed on mature granulocytes. Involved in IL-5 induced survival [66]

Data presented are fold change in gene expression

*COPD* chronic obstructive pulmonary disease, *FDR* false discovery rate

^a^Fold change in gene expression significant (FDR < 0.05) in both cohorts

^b^Fold change in gene expression significant by FDR in the asthma study and by *p* value in the COPD study

^c^Fold change in gene expression significant (FDR < 0.05) in the Phase IIb asthma study only

In the asthma cohort, Charcot-Leyden crystal galectin (CLC) revealed the most prominent downregulation in expression in the benralizumab-treated arm across all patients, as well as for eosinophil-high and eosinophil-low patients (> 4-fold, FDR < 0.05). IL-5Rα expression was significantly decreased for all patients, as well as for eosinophil-high and eosinophil-low patients in the benralizumab-treated group (FDR < 0.05). P2RY14, CD9, CD24, and FCER1A (Fc fragment of IgE receptor Ia) exhibited decreased expression across all patients treated with benralizumab (FDR < 0.05). However, this did not remain significantly altered when analyzed by eosinophil-high vs. eosinophil-low subgroups. An additional eight genes (CCL23, CEBPE, HES1, PTGDR2, SIGLEC8, SLC29A1, SMPD3, and SORD) were also significantly decreased in the eosinophil-high benralizumab treatment group. PRSS33 was among the genes demonstrating the most decrease in the benralizumab-treated arm across all patients and for both eosinophil-high and eosinophil-low groups.

In the COPD cohort, CLC provided the most prominent downregulation in expression in the benralizumab-treated arm across all patients (> 5-fold, FDR < 0.05) and for eosinophil-high (> 4-fold, raw *p* < 0.05, but not FDR) and eosinophil-low patients (> 1.5-fold, raw *p* < 0.05, but not FDR). IL-5Rα also exhibited significantly decreased expression for all patients (> 1.9-fold, FDR < 0.05) and for both eosinophil-high (> 2-fold, raw *p* < 0.05, but not FDR) and eosinophil-low (> 5-fold, FDR < 0.05) patients. Three additional genes, ADORA3, PRSS33, and OLIG2 were also significantly decreased by at least 1.5-fold in the benralizumab-treated group compared to placebo (FDR < 0.05). All three genes were decreased by more than 2-fold in the benralizumab-treated eosinophil-high patients (raw *p* < 0.05, but not FDR), while OLIG2 and PRSS33 were decreased by at least 1.5-fold for benralizumab-treated eosinophil-low patients (raw *p* < 0.05, but not FDR).

### Gene signatures

**Fig. 2:**
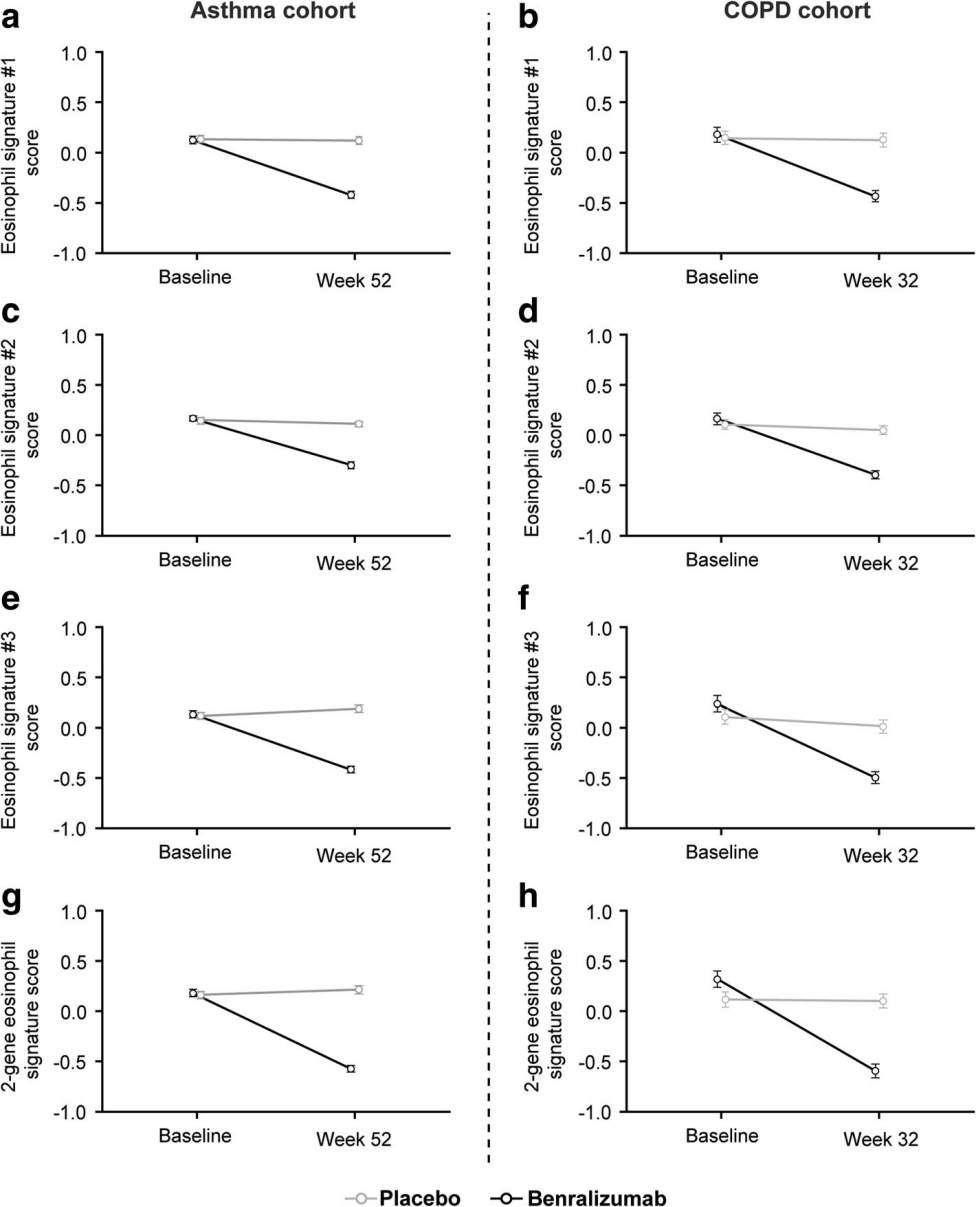
GSVA scores for eosinophil signatures in patients with asthma and COPD over time. GSVA signature scores of the four eosinophil-related gene signatures for the overall population in the asthma cohort treated with benralizumab vs. placebo (**a, c, e**, and **g**). GSVA signature scores of the four eosinophil-related gene signatures for the overall population in the COPD cohort treated with benralizumab vs. placebo (**b, d, f**, and **h**). Signature scores ranged from − 1 to 1, with negative scores indicating relative decreases in signature expression and positive scores indicating relative elevations in signature expression. Mean GSVA scores per signature are given for each treatment arm at each timepoint with standard error bars shown. *COPD* chronic obstructive pulmonary disease, *GSVA* gene set variation analysis

A list of the genes included in significantly altered signatures and the modifications in gene signatures in response to benralizumab treatment are provided in Table 3. Overall, any immune-related gene signatures altered in response to benralizumab treatment were downregulated (Table 3). All four eosinophil gene signatures (eosinophil signature #1, eosinophil signature #2, eosinophil signature #3, and 2-gene eosinophil signature) were significantly downregulated (FDR < 0.05) in response to benralizumab treatment in both the asthma and COPD cohorts (Fig. 2), even after adjustment for baseline eosinophil counts *post hoc* (Additional file 5: Table S2). In addition to these eosinophil signatures, mast cells and type I interferon signatures were also significantly downregulated (FDR < 0.05) in benralizumab- vs. placebo-treated patients in the asthma cohort, although the magnitude of reduction was small (Additional file 6: Figure S4).

**Table 3:** Modifications in gene signatures in response to benralizumab treatment

	Gene sets
Asthma cohort	
Upregulated gene sets	None
Downregulated gene sets	Interferon-related genes, eosinophil signature #1, eosinophil signature #2 [31], mast cell signature, 2-gene eosinophil signature, eosinophil signature #3 [32]
COPD cohort	
Upregulated gene sets	None
Downregulated gene sets	2-gene eosinophil signature, eosinophil signature #1, eosinophil signature #2 [31], eosinophil signature #3 [32]
2-gene eosinophil signature	*PRSS33*, *CCL23*
Eosinophil signature #1	*PRSS33*, *GPR44*, *CLC*, *ADORA3*, *IDO1*, *OLIG1*, *EMR1*, *CCR3*, *VSTM1*, *LGALS12*, *CAT*, *CAMK1*, *CD9*, *SIGLEC10*, *TKTL1*, *FLVCR1*, *P2RY14*, *LOC283070*, *FAM101B*
Eosinophil signature #2 [31]	*PRSS33*, *SIGLEC10*, *GPR82*, *PDE4D*, *PIK3R6*, *CAT*, *SORD*, *SLC29A1*, *MARK3*, *CASP3*, *EPN2*, *FGFR2*, *GADD45A*, *AREG*, *AREGB*, *CLC*, *PTGDR2*, *CYP4F12*, *IL1RL1*, *SIGLEC8*, *GFI1B*, *PAPSS1*, *MAX*, *ARHGEF6*, *RIPK2*, *CSF1*, *CACNA1D*, *GATA1*, *CCL23*, *IL5RA*, *RPL13P5*, *GSTM4*, *OLIG2*, *CEBPE*, *DAPK2*, *CYSLTR2*, *ARL6IP6*, *LINC00085*, *PYROXD2*, *EXOC3*, *SEMA7A*, *VSTM1*, *SLC16A14*, *EPN2-IT1*
Eosinophil signature #3 [32]	*CACNG6*, *CCL23*, *GPR44*, *HSD3B7*, *IDO1*, *SIGLEC8*
Interferon-related genes	*DNAPTP6*, *EPSTI1*, *HERC5*, *IFI27*, *IFI44*, *IFI44L*, *IFI6*, *IFIT1*, *IFIT3*, *ISG15*, *LAMP3*, *LY6E*, *MX1*, *OAS1*, *OAS2*, *OAS3*, *PLSCR1*, *RSAD2*, *RTP4*, *SIGLEC1*, *USP18*
Mast cell signature	*CPA3*, *TPSB2*, *TPSAB1*

Threshold for significance: FDR < 0.05

*COPD* chronic obstructive pulmonary disease, *FDR* false discovery rate

An assessment of signature differences in eosinophil-high and eosinophil-low patients found that the expression of only the four eosinophil signatures were consistently decreased in benralizumab- vs. placebo-treated patients in both subgroups of the asthma cohort. In contrast, for the eosinophil-high patients in the COPD cohort, only three of the four eosinophil signatures (eosinophil signature #1, eosinophil signature #2, and 2-gene eosinophil signature) were significantly decreased (FDR < 0.05) in benralizumab-treated patients. For the eosinophil-low patients in the COPD cohort, only two of the four eosinophil signatures (eosinophil signature #3 and 2-gene signature) were significantly decreased (FDR < 0.05). Gene signatures for plasma cells, B cells, and neutrophils were also investigated, but did not demonstrate any significant change.

### Discussion

Eosinophilic airway inflammation, typically associated with asthma, may also play an important role in COPD [8, 13]. Furthermore, recent advances in our understanding of the relationship between eosinophilic inflammation and the different characteristics of asthma and COPD have led to the development of new therapies targeting eosinophilic inflammation in these patient populations [33–35]. One such treatment, benralizumab, reduces sputum and blood eosinophil counts by enhanced antibody-dependent cell-mediated cytotoxicity, and has been reported to reduce exacerbations in patients with eosinophilic asthma [21, 24]. Moreover, depletion of eosinophils following benralizumab treatment also reduces eosinophil-resident cytotoxic granule proteins, including eosinophil cationic protein and eosinophil-derived neurotoxin [22]. Results in COPD have been inconsistent, with a Phase IIa study of patients with eosinophilic COPD demonstrating a numerical reduction in exacerbation rates [25], while two Phase III studies did not meet their primary endpoints [26, 27].

Our analysis sought to characterize the effects of benralizumab 100 mg Q8W SC on blood protein and gene expression and determine modulations in inflammatory markers and immune-related signaling pathways by proteomic and gene-expression analyses in patients with asthma and COPD. Results demonstrated that only two protein analytes, eotaxin-1 and eotaxin-2, were significantly upregulated following treatment with benralizumab in both the asthma and COPD cohorts, with greater upregulation being observed with eotaxin-1. The magnitude of upregulation of eotaxin-1 and eotaxin-2 was greater in eosinophil-high patients than eosinophil-low patients in both the asthma and COPD cohorts. Eotaxin-1 and eotaxin-2 are chemotactic cytokines that play a crucial role in eosinophil chemotaxis and recruitment of eosinophils to sites of inflammation in asthma and COPD [36]. In the airways, these chemokines increase the recruitment of inflammatory cells, especially eosinophils, and are associated with a more severe form of airway disease [37, 38]. Moreover, eotaxin-1 is secreted by the endothelial cells of the pulmonary artery, thereby increasing eosinophil survival [39]. Indeed, a study that measured cytokines in the lung lavage fluid and plasma of COPD patients to determine if the concentrations of T-cell or eosinophil-related cytokines were predictive of the future course of the disease, indicated that bronchial lavage concentrations of eotaxin-1 increased with disease severity. This suggested that the presence of eosinophils may be an indicator of the progression of COPD [40]. In contrast, plasma eotaxin-1 concentrations were significantly lower in stable COPD patients [40]. Increase in eotaxin-1 and eotaxin-2 concentrations in the serum of asthma patients following benralizumab treatment has been reported in two Phase I/IIa benralizumab studies [22]. In addition, similar findings have been suggested with the IL-5-neutralizing monoclonal antibody mepolizumab, as eosinophils demonstrated decreased responsiveness to increasing concentrations of eotaxin-1 and eotaxin-2 ex vivo and concentrations of IL-5 increased in vivo following treatment [41]. An increase in the concentration of other select serum cytokines following targeted reduction of receptor-expressing immune cell subtypes has also been observed. For example, following rituximab-mediated B-cell depletion, increased concentrations of B-cell activating factor of the tumor necrosis factor family were reported [42]. While the precise mechanism of upregulation of eotaxin-1 and eotaxin-2 following benralizumab treatment is unclear, one hypothesis is that an autoregulatory feedback mechanism may be involved following eosinophil depletion that may lead to increased production of these chemokines [22, 41].

BDNF, a protein that positively relates to eosinophil counts in atopic dermatitis [43, 44], was the only protein that was significantly decreased in the asthma cohort following benralizumab treatment. BDNF protein concentrations are upregulated in severe asthma patients and in those with airway hyperresponsiveness [45]. This suggests regulation by type-2 cytokines and a role in the pathophysiology of asthma. However, in this study, the magnitude of change of BDNF observed in benralizumab-treated patients compared with those treated with placebo was small and considered not to be pharmacologically relevant since the change was well within the baseline concentration range.

Benralizumab was also associated with a significant reduction in the expression of genes associated with eosinophils and basophils, with the magnitude of downregulation greater for eosinophil-high vs. eosinophil-low patients. This finding is not unexpected since treatment with benralizumab, as a result of its mechanism of action, directly depletes eosinophils and basophils in the peripheral blood circulation [22, 28]. The selectivity of this depletion with treatment was confirmed on a whole transcriptome level in the peripheral blood of asthma and COPD patients. Notably, CLC (a known marker of eosinophils) and IL-5Rα (selectively expressed on eosinophils and basophils), which are targeted by benralizumab, exhibited the most prominent reduction in expression for patients treated with benralizumab, as well as for eosinophil-high and eosinophil-low patients. This further demonstrates the inhibitory effect of benralizumab on eosinophilic inflammation. While expression of some of the genes that were decreased were not specific markers of eosinophils or basophils, they were all associated with eosinophil or basophil biology [46–66].

Gene signature analysis further confirmed the selectivity of action of benralizumab, with significant decreases in all four eosinophil-related gene signatures observed for the benralizumab-treated group, across all asthma and COPD patients, and for eosinophil-high and eosinophil-low patients. In addition, expression of a set of interferon-related genes exhibited decreased expression for all patients treated with benralizumab. However, the magnitude of this reduction was much lower than that observed for the eosinophil gene signatures and may not be biologically relevant. Relative to the effects on the eosinophil signatures, we also detected a small but significant decrease in mast cell gene signatures in patients treated with benralizumab compared with those treated with placebo. Based on in-vitro studies, mast cells are suggested to express IL-5Rα [67]. However, there is limited direct in-vivo evidence that mast cells express IL-5Rα in humans. Although IL-5Rα expression has been detected on mast cells in the bone marrow of patients with mastocytosis using flow cytometry [68], IL-5Rα expression was not observed on mast cells in the lung tissue of asthma patients using immunohistochemistry [21]. While we do not have any data to demonstrate that IL-5Rα expression is associated with the mast cell gene signatures in this study, we hypothesize that the observed decrease in mast cell signature may have been the result of a subpopulation of mast cells in blood that express IL-5Rα.

Our study has several limitations. First, the analysis on the COPD cohort was underpowered compared with the asthma cohort, owing to the smaller patient population, which meant that it was not adequately powered to determine statistically significant differences between blood eosinophil-high and eosinophil-low subgroups using more stringent FDR thresholds. Secondly, since this analysis was based on blood samples, it may not entirely reflect the effect of benralizumab within local tissues and the airways. However, this study was unique in that it examined patients with eosinophilic disease in both asthma and COPD settings, thereby demonstrating the pharmacologic activity of benralizumab in both disease states and demonstrating that there are common effects of treatment with benralizumab in patients with eosinophilic airway disease. Analysis of the blood transcriptome and a large panel of protein analytes confirmed the selectivity of action of benralizumab since protein and gene-related immune signaling pathways and immune cell markers, other than eosinophils and basophils, were unchanged post-treatment.

These results suggest that benralizumab is highly selective, affecting the expression of proteins and genes specifically associated with eosinophils or basophils, and that this effect is most prominent in patients with high baseline blood eosinophil counts.

## Conclusions

Benralizumab is highly selective, modulating proteins or genes associated with eosinophils or basophils in the blood of both patients with asthma and COPD. Modulated protein and gene expression patterns are most prominently altered in patients with high blood eosinophilia (≥300 cells/μL) compared with those with low blood eosinophilia (< 300 cells/μL).

## Additional files

Additional file 1: **Figure S1.** Study designs for the asthma and COPD studies [24, 25]**.** COPD, chronic obstructive pulmonary disease; SC, subcutaneous. (TIF 2254 kb)

Additional file 2: **Table S1.** Protein analyte concentrations for benralizumab- and placebo-treated patients with asthma and COPD. (DOCX 84 kb)

Additional file 3: **Figure S2.** Protein analyte concentrations of eotaxin-1 and eotaxin-2 by eosinophil-high and eosinophil-low patient groups**.** Concentrations of eotaxin-1 and eotaxin-2 at baseline and after 52 weeks of treatment with benralizumab vs. placebo in EOS-high and EOS-low patients in the asthma cohort (a, c, e, and g). Concentrations of eotaxin-1 and eotaxin-2 at baseline and after 32 weeks of treatment with benralizumab vs. placebo for EOS-high and EOS-low patients in the COPD cohort (b, d, f, and h). Boxplots display the 25th–75th percentile values, with bars denoting median values. Boxes are labeled with the mean concentration per treatment arm. The dotted line denotes the analyte LLOQ. COPD, chronic obstructive pulmonary disease; EOS, eosinophils; EOS-high, eosinophil count ≥300 cells/μL; EOS-low, eosinophil count < 300 cells/μL; LLOQ, lower limit of quantification. (TIF 2435 kb)

Additional file 4: **Figure S3.** Protein analyte concentrations of BDNF across all patients with asthma**.** BDNF protein concentrations after 52 weeks of treatment with benralizumab vs. placebo. Boxplots display the 25th–75th percentile values, with bars denoting median values. Boxes are labeled with the mean concentration per treatment arm. The dotted line denotes the analyte LLOQ. BDNF, brain-derived neurotrophic factor; FDR, false discovery rate; LLOQ, lower limit of quantification. (TIF 865 kb)

Additional file 5: **Table S2.** *Post-hoc* analyses: effect of baseline blood eosinophil counts on eosinophil gene signatures. (DOCX 24 kb)

Additional file 6: **Figure S4.** GSVA scores for (a) interferon-related signature and (b) mast cell signature in patients with asthma**.** GSVA scores are given for internally defined type 1 interferon-related gene signature and internally defined mast cell gene signature assessed across asthma patients in benralizumab-treated or placebo arms. Mean GSVA scores per signature are given for each treatment arm at each time point with standard error bars. Signature scores ranged from − 1 to 1, with negative scores indicating relative decreases in signature expression and positive scores indicating relative elevations in signature expression. GSVA, gene set variation analysis. (TIF 435 kb)

## Abbreviations

ANOVA: Analysis of variance
BDNF: Brain-derived neurotrophic factor
CLC: Charcot-Leyden crystal galectin
COPD: Chronic obstructive pulmonary disease
ELEN index: Eosinophil/lymphocyte and eosinophil/neutrophil combined ratio index
FDR: False discovery rate
GSVA: Gene set variation analysis
ICS: Inhaled corticosteroids
IL-5Rα: Interleukin-5 receptor alpha subunit
LLOQ: Lower limit of quantification
Q4W: Dosing every 4 weeks
Q8W: Dosing every 8 weeks
SC: Subcutaneous

## References

[1] Benton MJ, Lim TK, Ko FWS, Kan OK, Mak JCW (2018). Year in review 2017: Chronic obstructive pulmonary disease and asthma. Respirology..

[2] Peters SP, Ferguson G, Deniz Y, Reisner C (2006). Uncontrolled asthma: a review of the prevalence, disease burden and options for treatment. Respir Med.

[3] Halpin DM, Miravitlles M, Metzdorf N, Celli B (2017). Impact and prevention of severe exacerbations of COPD: a review of the evidence. Int J Chron Obstruct Pulmon Dis..

[4] Nurmagambetov T, Kuwahara R, Garbe P (2018). The economic burden of asthma in the United States, 2008–2013. Ann Am Thorac Soc..

[5] Global Strategy for the Diagnosis, Management and Prevention of COPD, Global Initiative for Chronic Obstructive Lung Disease (GOLD) 2018. http://www.goldcopd.org/. Accessed 21 Mar 2018.

[6] Turner AM, Tamasi L, Schleich F, Hoxha M, Horvath I, Louis R (2015). Clinically relevant subgroups in COPD and asthma. Eur Respir Rev.

[7] Heaney LG, McGarvey LP (2017). Personalised medicine for asthma and chronic obstructive pulmonary disease. Respiration.

[8] Zhang JY, Wenzel SE (2007). Tissue and BAL based biomarkers in asthma. Immunol Allergy Clin N Am.

[9] Price D, Wilson AM, Chisholm A, Rigazio A, Burden A, Thomas M (2016). Predicting frequent asthma exacerbations using blood eosinophil count and other patient data routinely available in clinical practice. J Asthma Allergy.

[10] Garcia G, Taille C, Laveneziana P, Bourdin A, Chanez P, Humbert M (2013). Anti-interleukin-5 therapy in severe asthma. Eur Respir Rev.

[11] Hospers JJ, Schouten JP, Weiss ST, Postma DS, Rijcken B (2000). Eosinophilia is associated with increased all-cause mortality after a follow-up of 30 years in a general population sample. Epidemiology.

[12] Talini D, Novelli F, Bacci E, Bartoli M, Cianchetti S, Costa F (2015). Sputum eosinophilia is a determinant of FEV1 decline in occupational asthma: results of an observational study. BMJ Open.

[13] Kolsum U, Ravi A, Hitchen P, Maddi S, Southworth T, Singh D (2017). Clinical characteristics of eosinophilic COPD versus COPD patients with a history of asthma. Respir Res.

[14] Siva R, Green RH, Brightling CE, Shelley M, Hargadon B, McKenna S (2007). Eosinophilic airway inflammation and exacerbations of COPD: a randomised controlled trial. Eur Respir J.

[15] Woodruff PG, Modrek B, Choy DF, Jia G, Abbas AR, Ellwanger A (2009). T-helper type 2-driven inflammation defines major subphenotypes of asthma. Am J Respir Crit Care Med.

[16] Chung KF, Wenzel SE, Brozek JL, Bush A, Castro M, Sterk PJ (2014). International ERS/ATS guidelines on definition, evaluation and treatment of severe asthma. Eur Respir J.

[17] Wenzel SE, Covar R (2006). Update in asthma 2005. Am J Respir Crit Care Med.

[18] Pavord ID, Chanez P, Criner GJ, Kerstjens HAM, Korn S, Lugogo N (2017). Mepolizumab for eosinophilic chronic obstructive pulmonary disease. N Engl J Med.

[19] McEvoy CE, Niewoehner DE (1997). Adverse effects of corticosteroid therapy for COPD. Chest.

[20] Heffler E, Nascimento Girardi Madeira L, Ferrando M, Puggioni F, Racca F, Malvezzi L, et al. Inhaled Corticosteroids Safety and Adverse Effects in Patients with Asthma. J Allergy Clin Immunol Pract. 2018; doi:10.1016/j.jaip.2018.01.025. 10.1016/j.jaip.2018.01.025 29408385

[21] Kolbeck R, Kozhich A, Koike M, Peng L, Andersson CK, Damschroder MM (2010). MEDI-563, a humanized anti-IL-5 receptor alpha mAb with enhanced antibody-dependent cell-mediated cytotoxicity function. J Allergy Clin Immunol.

[22] Pham TH, Damera G, Newbold P, Ranade K (2016). Reductions in eosinophil biomarkers by benralizumab in patients with asthma. Respir Med.

[23] FASENRA™ (benralizumab) prescribing information. November 2017. https://www.accessdata.fda.gov/drugsatfda_docs/label/2017/761070s000lbl.pdf. Accessed 04 Apr 2018.

[24] Castro M, Wenzel SE, Bleecker ER, Pizzichini E, Kuna P, Busse WW (2014). Benralizumab, an anti-interleukin 5 receptor alpha monoclonal antibody, versus placebo for uncontrolled eosinophilic asthma: a phase 2b randomised dose-ranging study. Lancet Respir Med.

[25] Brightling CE, Bleecker ER, Panettieri RA, Bafadhel M, She D, Ward CK (2014). Benralizumab for chronic obstructive pulmonary disease and sputum eosinophilia: a randomised, double-blind, placebo-controlled, phase 2a study. Lancet Respir Med.

[26] AstraZeneca News Release. “AstraZeneca provides update on GALATHEA Phase III trial for Fasenra in chronic obstructive pulmonary disease.” https://www.astrazeneca.com/content/astraz/media-centre/press-releases/2018/astrazeneca-provides-update-on-galathea-phase-iii-trial-for-fasenra-in-chronic-obstructive-pulmonary-disease-11052018.html. Accessed 13 June 2018.

[27] AstraZeneca News Release. “Update on TERRANOVA Phase III trial for Fasenra in chronic obstructive pulmonary disease.” https://www.astrazeneca.com/content/astraz/media-centre/press-releases/2018/update-on-terranova-phase-iii-trial-for-fasenra-in-chronic-obstructive-pulmonary-disease-30052018.html. Accessed 13 June 2018.

[28] Eck S, Castro M, Sinibaldi D, White W, Folliot K, Gossage D (2014). Benralizumab effect on blood basophil counts in adults with uncontrolled asthma. Eur Respir J.

[29] Khatry DB, Gossage DL, Geba GP, Parker JM, Jarjour NN, Busse WW (2015). Discriminating sputum-eosinophilic asthma: accuracy of cutoffs in blood eosinophil measurements versus a composite index. ELEN J Allergy Clin Immunol.

[30] Hanzelmann S, Castelo R, Guinney J (2013). GSVA: gene set variation analysis for microarray and RNA-seq data. BMC Bioinformatics.

[31] Allantaz F, Cheng DT, Bergauer T, Ravindran P, Rossier MF, Ebeling M (2012). Expression profiling of human immune cell subsets identifies miRNA-mRNA regulatory relationships correlated with cell type specific expression. PLoS One.

[32] Choy DF, Jia G, Abbas AR, Morshead KB, Lewin-Koh N, Dua R (2016). Peripheral blood gene expression predicts clinical benefit from anti-IL-13 in asthma. J Allergy Clin Immunol.

[33] Tashkin DP, Wechsler ME (2018). Role of eosinophils in airway inflammation of chronic obstructive pulmonary disease. Int J Chron Obstruct Pulmon Dis.

[34] Kostikas K, Brindicci C, Patalano F. Blood eosinophils as biomarkers to drive treatment choices in asthma and COPD. Curr Drug Targets. 2018. 10.2174/1389450119666180212120012. 10.2174/1389450119666180212120012 PMC6225326 29437007

[35] Nixon J, Newbold P, Mustelin T, Anderson GP, Kolbeck R (2017). Monoclonal antibody therapy for the treatment of asthma and chronic obstructive pulmonary disease with eosinophilic inflammation. Pharmacol Ther.

[36] Paplinska M, Hermanowicz-Salamon J, Nejman-Gryz P, Bialek-Gosk K, Rubinsztajn R, Arcimowicz M (2012). Expression of eotaxins in the material from nasal brushing in asthma, allergic rhinitis and COPD patients. Cytokine.

[37] Dent G, Hadjicharalambous C, Yoshikawa T, Handy RL, Powell J, Anderson IK (2004). Contribution of eotaxin-1 to eosinophil chemotactic activity of moderate and severe asthmatic sputum. Am J Respir Crit Care Med.

[38] Scheicher ME, Teixeira MM, Cunha FQ, Teixeira AL, Filho JT, Vianna EO (2007). Eotaxin-2 in sputum cell culture to evaluate asthma inflammation. Eur Respir J.

[39] Farahi N, Cowburn AS, Upton PD, Deighton J, Sobolewski A, Gherardi E (2007). Eotaxin-1/CC chemokine ligand 11: a novel eosinophil survival factor secreted by human pulmonary artery endothelial cells. J Immunol.

[40] D'Armiento JM, Scharf SM, Roth MD, Connett JE, Ghio A, Sternberg D (2009). Eosinophil and T cell markers predict functional decline in COPD patients. Respir Res.

[41] Stein ML, Villanueva JM, Buckmeier BK, Yamada Y, Filipovich AH, Assa'ad AH (2008). Anti-IL-5 (mepolizumab) therapy reduces eosinophil activation ex vivo and increases IL-5 and IL-5 receptor levels. J Allergy Clin Immunol.

[42] Lavie F, Miceli-Richard C, Ittah M, Sellam J, Gottenberg JE, Mariette X (2007). Increase of B cell-activating factor of the TNF family (BAFF) after rituximab treatment: insights into a new regulating system of BAFF production. Ann Rheum Dis.

[43] Folster-Holst R, Papakonstantinou E, Rudrich U, Buchner M, Pite H, Gehring M (2016). Childhood atopic dermatitis-brain-derived neurotrophic factor correlates with serum eosinophil cationic protein and disease severity. Allergy.

[44] Namura K, Hasegawa G, Egawa M, Matsumoto T, Kobayashi R, Yano T (2007). Relationship of serum brain-derived neurotrophic factor level with other markers of disease severity in patients with atopic dermatitis. Clin Immunol.

[45] Watanabe T, Fajt ML, Trudeau JB, Voraphani N, Hu H, Zhou X (2015). Brain-derived neurotrophic factor expression in asthma. Association with severity and type 2 inflammatory processes. Am J Respir Cell Mol Biol.

[46] Calafat J, Janssen H, Knol EF, Weller PF, Egesten A (1997). Ultrastructural localization of Charcot-Leyden crystal protein in human eosinophils and basophils. Eur J Haematol.

[47] Ackerman SJ, Gleich GJ, Weller PF, Ottesen EA (1981). Eosinophilia and elevated serum levels of eosinophil major basic protein and Charcot-Leyden crystal protein (lysophospholipase) after treatment of patients with Bancroft's filariasis. J Immunol.

[48] Ackerman SJ, Weil GJ, Gleich GJ (1982). Formation of Charcot-Leyden crystals by human basophils. J Exp Med.

[49] Golightly LM, Thomas LL, Dvorak AM, Ackerman SJ (1992). Charcot-Leyden crystal protein in the degranulation and recovery of activated basophils. J Leukoc Biol.

[50] Toyama S, Okada N, Matsuda A, Morita H, Saito H, Fujisawa T (2017). Human eosinophils constitutively express a unique serine protease, PRSS33. Allergol Int.

[51] Hwang SM, Uhm TG, Lee SK, Kong SK, Jung KH, Binas B (2016). Olig2 is expressed late in human eosinophil development and controls Siglec-8 expression. J Leukoc Biol.

[52] Cheng YX, Foster B, Holland SM, Klion AD, Nutman TB, Casale TB (2006). CD2 identifies a monocyte subpopulation with immunoglobulin E-dependent, high-level expression of Fc epsilon RI. Clin Exp Allergy..

[53] Oda T, Morikawa N, Saito Y, Masuho Y, Matsumoto S (2000). Molecular cloning and characterization of a novel type of histamine receptor preferentially expressed in leukocytes. J Biol Chem.

[54] Ponath PD, Qin S, Post TW, Wang J, Wu L, Gerard NP (1996). Molecular cloning and characterization of a human eotaxin receptor expressed selectively on eosinophils. J Exp Med.

[55] Uguccioni M, Mackay CR, Ochensberger B, Loetscher P, Rhis S, LaRosa GJ (1997). High expression of the chemokine receptor CCR3 in human blood basophils. Role in activation by eotaxin, MCP-4, and other chemokines. J Clin Invest.

[56] Takatsu K, Takaki S, Hitoshi Y (1994). Interleukin-5 and its receptor system: implications in the immune system and inflammation. Adv Immunol.

[57] Toba K, Koike T, Shibata A, Hashimoto S, Takahashi M, Masuko M (1999). Novel technique for the direct flow cytofluorometric analysis of human basophils in unseparated blood and bone marrow, and the characterization of phenotype and peroxidase of human basophils. Cytometry.

[58] Yamada T, Sun Q, Zeibecoglou K, Bungre J, North J, Kay AB (1998). IL-3, IL-5, granulocyte-macrophage colony-stimulating factor receptor alpha-subunit, and common beta-subunit expression by peripheral leukocytes and blood dendritic cells. J Allergy Clin Immunol.

[59] Odemuyiwa SO, Ghahary A, Li Y, Puttagunta L, Lee JE, Musat-Marcu S (2004). Cutting edge: human eosinophils regulate T cell subset selection through indoleamine 2,3-dioxygenase. J Immunol.

[60] Ferreira MA, Jansen R, Willemsen G, Penninx B, Bain LM, Vicente CT (2017). Gene-based analysis of regulatory variants identifies 4 putative novel asthma risk genes related to nucleotide synthesis and signaling. J Allergy Clin Immunol.

[61] Esnault S, Kelly EA, Schwantes EA, Liu LY, DeLain LP, Hauer JA (2013). Identification of genes expressed by human airway eosinophils after an in vivo allergen challenge. PLoS One.

[62] Brown RA, Spina D, Page CP (2008). Adenosine receptors and asthma. Br J Pharmacol.

[63] Reeves JJ, Harris CA, Hayes BP, Butchers PR, Sheehan MJ (2000). Studies on the effects of adenosine A3 receptor stimulation on human eosinophils isolated from non-asthmatic or asthmatic donors. Inflamm Res.

[64] Kim JT, Gleich GJ, Kita H (1997). Roles of CD9 molecules in survival and activation of human eosinophils. J Immunol.

[65] Turk J, Maas RL, Brash AR, Roberts LJ, Oates JA (1982). Arachidonic acid 15-lipoxygenase products from human eosinophils. J Biol Chem.

[66] Temple R, Allen E, Fordham J, Phipps S, Schneider HC, Lindauer K (2001). Microarray analysis of eosinophils reveals a number of candidate survival and apoptosis genes. Am J Respir Cell Mol Biol.

[67] Dahl C, Hoffmann HJ, Saito H, Schiøtz PO (2004). Human mast cells express receptors for IL-3, IL-5 and GM-CSF; a partial map of receptors on human mast cells cultured in vitro. Allergy.

[68] Wilson TM, Maric I, Shukla J, Brown M, Santos C, Simakova O (2011). IL-5 receptor α levels in patients with marked eosinophilia or mastocytosis. J Allergy Clin Immunol.

