# Supplementary figures and images for: Modulation of blood inflammatory markers by benralizumab in patients with eosinophilic airway diseases
Source: Respir Res. 2019 Jan 18;20:14. doi: 10.1186/s12931-018-0968-8 (PMC6339432; doi:10.1186/s12931-018-0968-8)

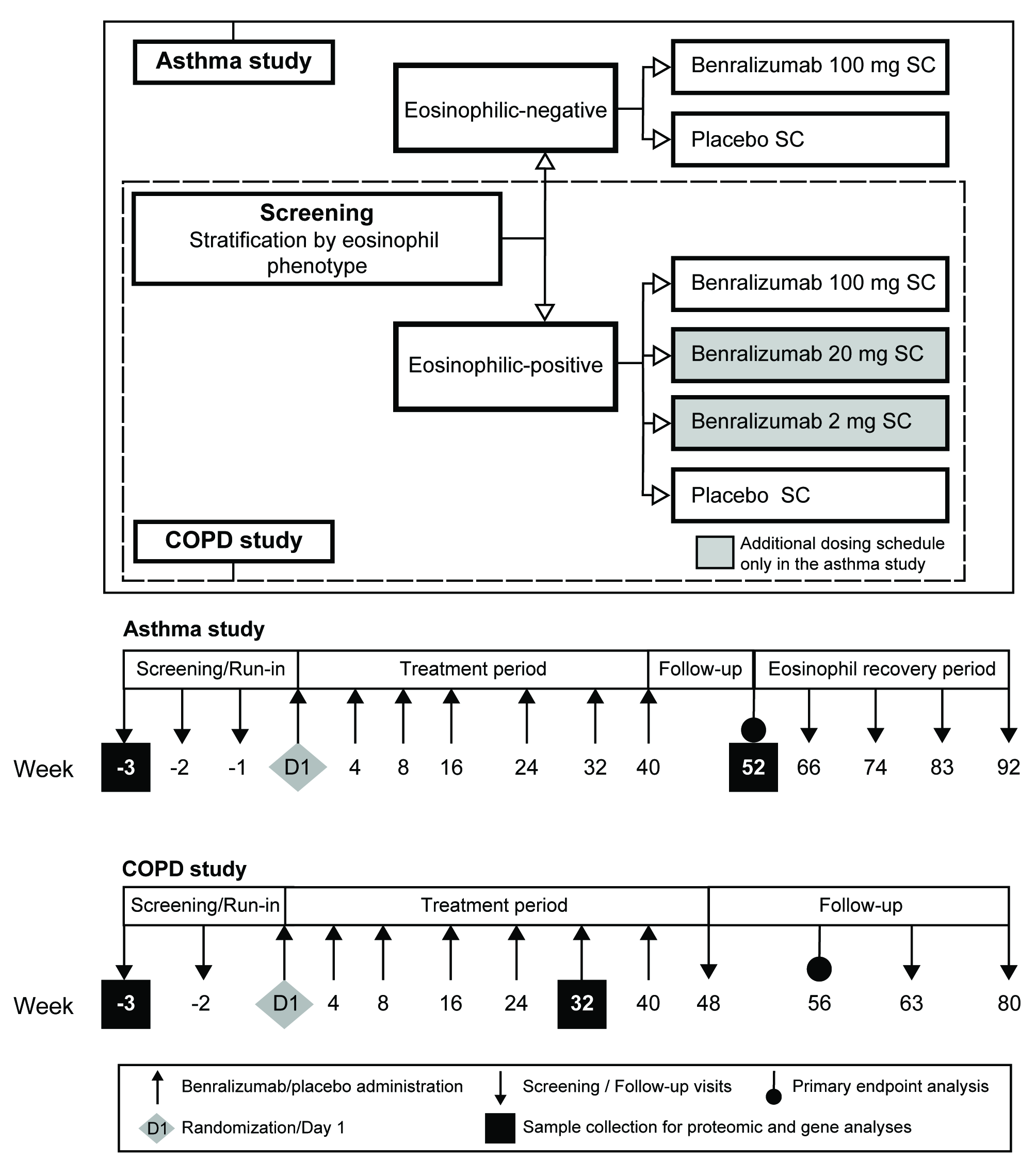

Supplement: Supplementary file 1 — Figure S1. Study designs for the asthma and COPD studies [24, 25]. COPD, chronic obstructive pulmonary disease; SC, subcutaneous. (TIF 2254 kb) [file 12931_2018_968_MOESM1_ESM.tif]

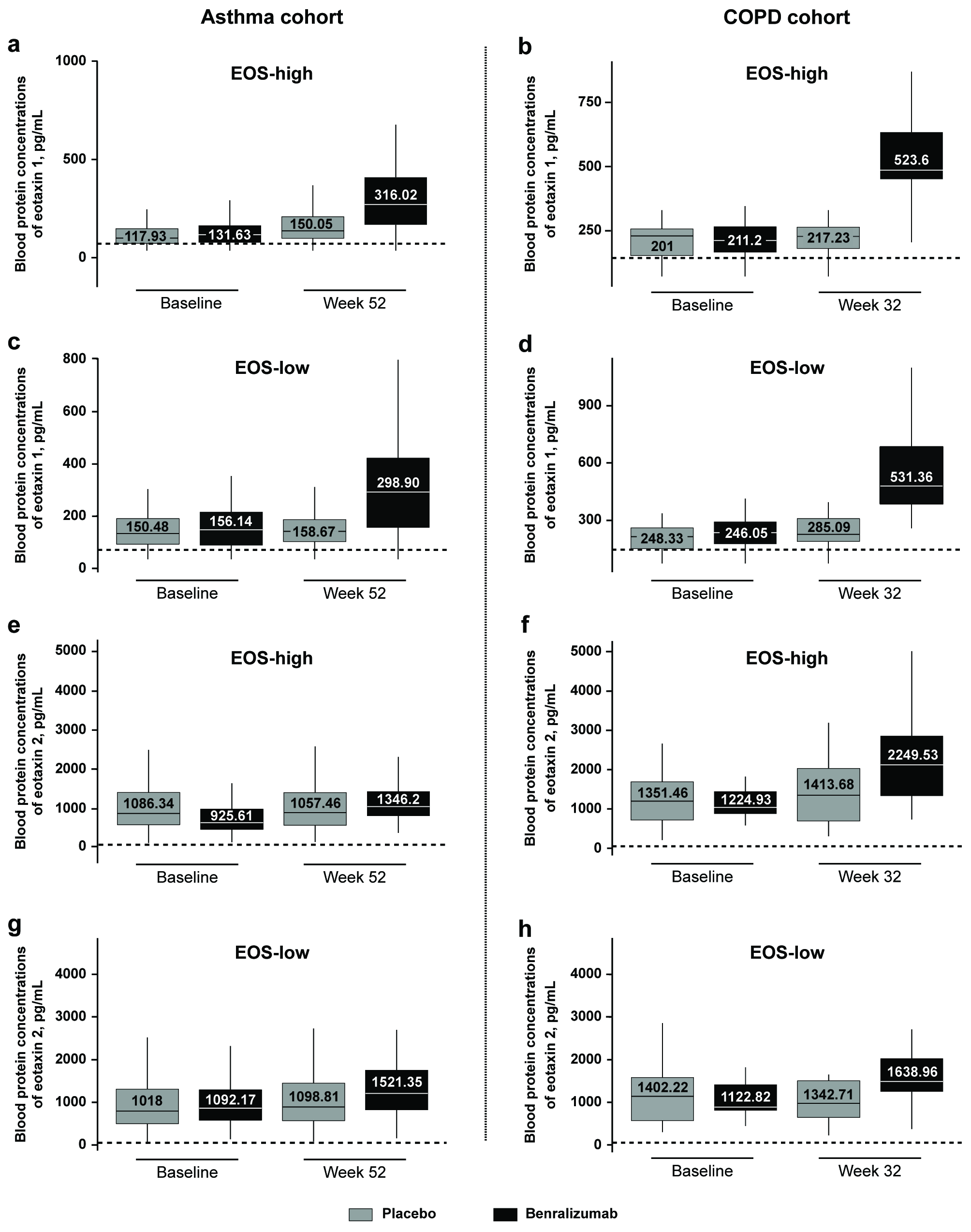

Supplement: Supplementary file 3 — Figure S2. Protein analyte concentrations of eotaxin-1 and eotaxin-2 by eosinophil-high and eosinophil-low patient groups. Concentrations of eotaxin-1 and eotaxin-2 at baseline and after 52 weeks of treatment with benralizumab vs. placebo in EOS-high and EOS-low patients in the asthma cohort (a, c, e, and g). Concentrations of eotaxin-1 and eotaxin-2 at baseline and after 32 weeks of treatment with benralizumab vs. placebo for EOS-high and EOS-low patients in the COPD cohort (b, d, f, and h). Boxplots display the 25th–75th percentile values, with bars denoting median values. Boxes are labeled with the mean concentration per treatment arm. The dotted line denotes the analyte LLOQ. COPD, chronic obstructive pulmonary disease; EOS, eosinophils; EOS-high, eosinophil count ≥300 cells/μL; EOS-low, eosinophil count < 300 cells/μL; LLOQ, lower limit of quantification. (TIF 2435 kb) [file 12931_2018_968_MOESM3_ESM.tif]

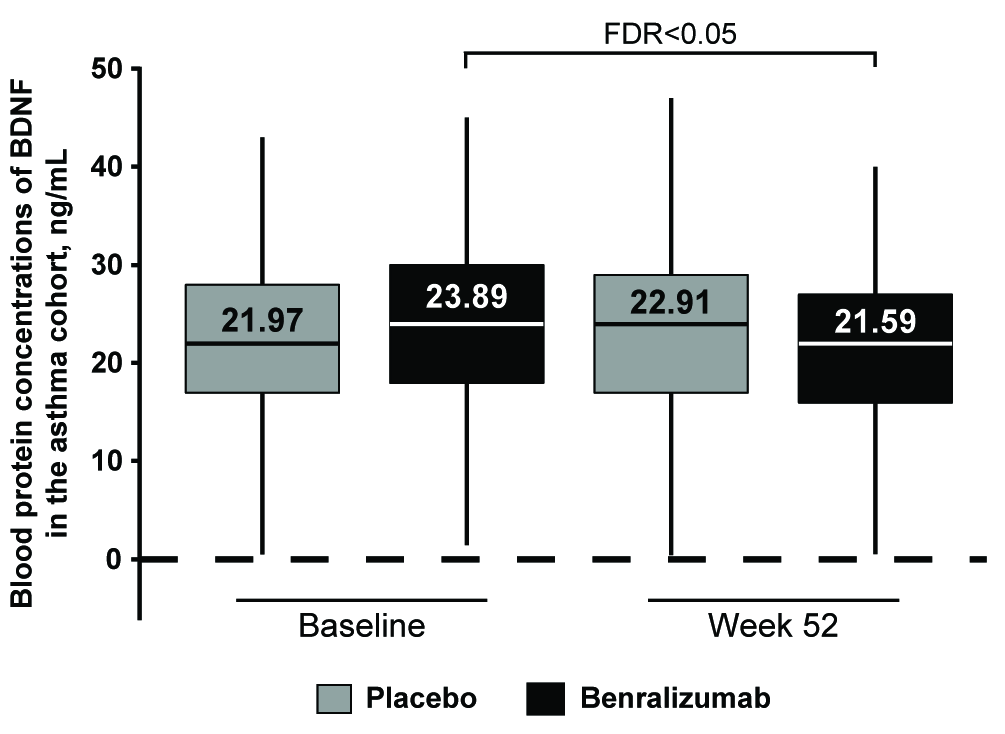

Supplement: Supplementary file 4 — Figure S3. Protein analyte concentrations of BDNF across all patients with asthma. BDNF protein concentrations after 52 weeks of treatment with benralizumab vs. placebo. Boxplots display the 25th–75th percentile values, with bars denoting median values. Boxes are labeled with the mean concentration per treatment arm. The dotted line denotes the analyte LLOQ. BDNF, brain-derived neurotrophic factor; FDR, false discovery rate; LLOQ, lower limit of quantification. (TIF 865 kb) [file 12931_2018_968_MOESM4_ESM.tif]

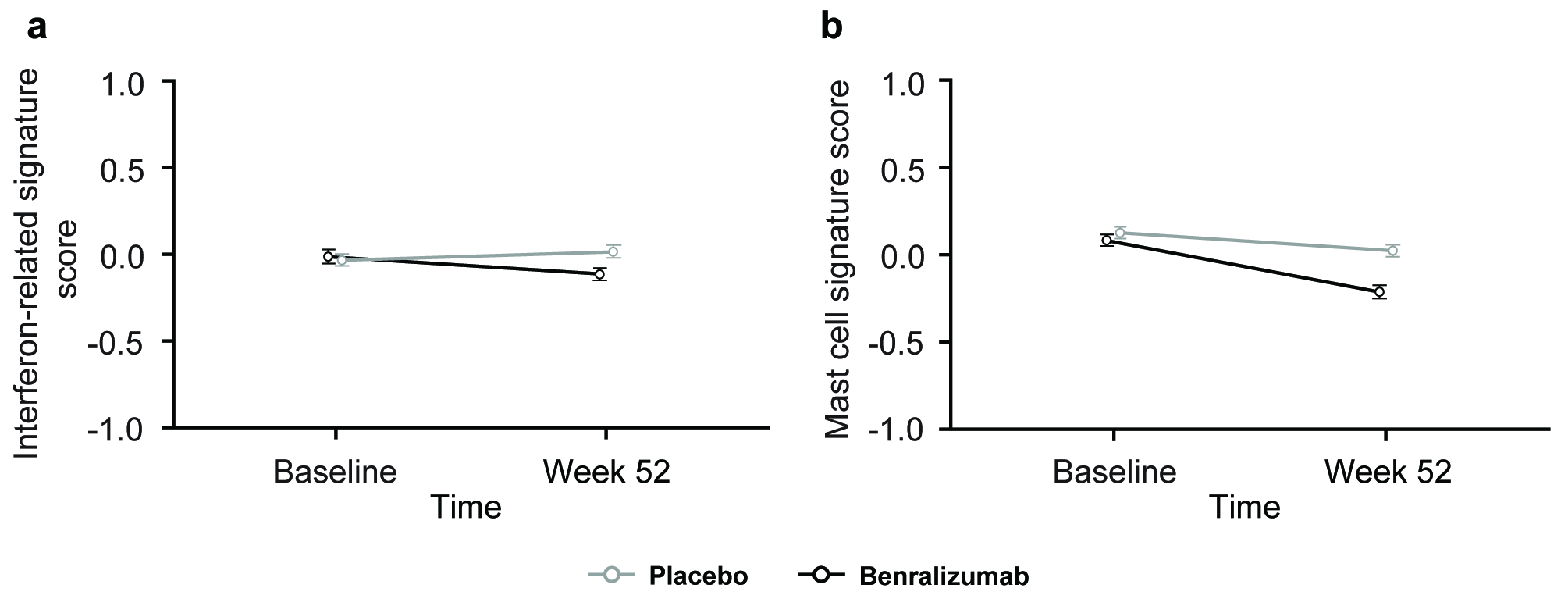

Supplement: Supplementary file 6 — Figure S4. GSVA scores for (a) interferon-related signature and (b) mast cell signature in patients with asthma. GSVA scores are given for internally defined type 1 interferon-related gene signature and internally defined mast cell gene signature assessed across asthma patients in benralizumab-treated or placebo arms. Mean GSVA scores per signature are given for each treatment arm at each time point with standard error bars. Signature scores ranged from − 1 to 1, with negative scores indicating relative decreases in signature expression and positive scores indicating relative elevations in signature expression. GSVA, gene set variation analysis. (TIF 435 kb) [file 12931_2018_968_MOESM6_ESM.tif]
