# Supplementary material for: Modulation of blood inflammatory markers by benralizumab in patients with eosinophilic airway diseases
Source: Respir Res. 2019 Jan 18;20:14. doi: 10.1186/s12931-018-0968-8 (PMC6339432; doi:10.1186/s12931-018-0968-8)
Supplement: Supplementary file 2 — Table S1. Protein analyte concentrations for benralizumab- and placebo-treated patients with asthma and COPD. (DOCX 84 kb) [file 12931_2018_968_MOESM2_ESM.docx]

**Table S1** Protein analyte concentrations for benralizumab- and placebo-treated patients with asthma and COPD

|  | **Asthma Cohort** | | | | **COPD cohort** | | | |
| --- | --- | --- | --- | --- | --- | --- | --- | --- |
|  | **Placebo baseline** | **Placebo Week 52** | **Benralizumab baseline** | **Benralizumab Week 52** | **Placebo baseline** | **Placebo Week 32** | **Benralizumab baseline** | **Benralizumab Week 32** |
| 6Ckine, pg/mL | 337.94 (99.41) | 361.72 (129.58) | 350.28 (101.76) | 370.3 (122.59) | 753.85 (266.35) | 694.93 (247.24) | 687.3 (284.36) | 616.47 (199.59) |
| A2Macro, mg/mL | 1.81 (0.4) | 1.8 (0.42) | 1.82 (0.43) | 1.79 (0.38) | 1.77 (0.77) | 1.6 (0.46) | 1.86 (0.66) | 1.67 (0.51) |
| AAT, mg/mL |  |  |  |  | 1.66 (0.52) | 1.77 (0.54) | 1.69 (0.3) | 1.79 (0.36) |
| Adiponectin, μg/mL | 4.64 (2.71) | 4.69 (2.72) | 4.77 (2.74) | 4.69 (2.65) | 5.61 (3.24) | 6.06 (3.71) | 6.43 (5.17) | 6.65 (5.38) |
| AgRP, pg/mL | 79.14 (11.42) | 78 (0) | 83.56 (35.85) | 80.91 (23.09) | 78 (0) | 82.37 (27.95) | 88.81 (49.54) | 84.02 (27.62) |
| ALP, ng/mL |  |  |  |  | 35.98 (6.93) | 37.63 (8) | 37.07 (11.54) | 38.81 (10.41) |
| ANG 1, ng/mL |  |  |  |  | 41.25 (16.86) | 45.73 (18.93) | 42.23 (15.41) | 41.79 (15.14) |
| ANG 2, ng/mL |  |  |  |  | 4.6 (1.71) | 4.28 (1.45) | 4.27 (1.91) | 4.08 (1.39) |
| Angiogenin, ng/mL |  |  |  |  | 441.88 (114.49) | 453.39 (112.55) | 428.91 (109.49) | 448.84 (140.55) |
| Apo D, μg/mL |  |  |  |  | 137.66 (46.66) | 147.37 (52.69) | 147 (42.32) | 140.63 (39.74) |
| Apo E, μg/mL |  |  |  |  | 39.54 (16.45) | 43.2 (23.47) | 47.37 (18.45) | 48.37 (19.72) |
| AR, pg/mL |  |  |  |  | 134 (0) | 134 (0) | 130.91 (20.28) | 130.91 (20.28) |
| AXL, ng/mL | 10.37 (3.42) | 10.93 (3.73) | 10.92 (3.59) | 11.11 (3.37) |  |  |  |  |
| B2M, μg/mL | 1.45 (0.44) | 1.47 (0.41) | 1.51 (0.95) | 1.45 (0.34) | 1.74 (0.75) | 2.07 (0.88) | 1.68 (0.51) | 2.11 (0.88) |
| BAFF, pg/mL | 648.78 (237.93) | 658.14 (231.71) | 645.86 (192.43) | 623.18 (185.63) | 918.73 (234.95) | 900.39 (209.72) | 970.99 (324.24) | 994 (301.89) |
| BDNF, ng/mL | 21.97 (8.83) | 22.91 (9.32) | 23.89 (9.16) | 21.59 (9.08) | 20.76 (8.11) | 21.51 (5.51) | 20.29 (5.71) | 20.24 (6.55) |
| BLC, pg/mL |  |  |  |  | 32.9 (8.51) | 35.51 (16.95) | 32.7 (7.78) | 33.44 (9.06) |
| BTC, pg/mL | 55.37 (5.23) | 55 (0) | 57.7 (26.48) | 55 (0) | 55 (0) | 55 (0) | 61.26 (41.02) | 56.79 (11.74) |
| C3, mg/mL |  |  |  |  | 1.22 (0.28) | 1.2 (0.26) | 1.22 (0.21) | 1.24 (0.22) |
| CA 15-3, Units/mL | 3.31 (1.72) | 3.54 (1.81) | 3.49 (1.89) | 3.73 (2.18) | 4.36 (2) | 4.53 (2.03) | 4.59 (2.26) | 4.8 (2.13) |
| CA9, ng/mL |  |  |  |  | 0.11 (0.05) | 0.11 (0.07) | 0.12 (0.06) | 0.13 (0.09) |
| Cathepsin D, ng/mL |  |  |  |  | 477.19 (142.78) | 505.61 (159.17) | 464.86 (132.5) | 517.12 (178.71) |
| CD40, ng/mL | 0.79 (0.2) | 0.83 (0.21) | 0.8 (0.2) | 0.82 (0.21) | 0.98 (0.23) | 1.01 (0.26) | 1.01 (0.52) | 1.08 (0.59) |
| CD40-L, ng/mL | 1.76 (1.14) | 1.78 (1.21) | 1.81 (1.16) | 1.75 (1.22) | 1.93 (1.17) | 2.05 (1.23) | 2.6 (1.97) | 2.31 (1.36) |
| CEACAM1, ng/mL |  |  |  |  | 15.8 (4.67) | 17.55 (5.31) | 17.88 (6.72) | 18.73 (6.38) |
| CgA, ng/mL |  |  |  |  | 594.66 (443.31) | 637.41 (382.95) | 608.37 (452.83) | 724.79 (564.48) |
| CK-MB, ng/mL | 1.43 (2.59) | 1.3 (1.3) | 1.25 (1.42) | 1.43 (3.02) | 2.2 (1.24) | 2.59 (1.97) | 2.15 (1.54) | 2.38 (1.86) |
| CLU, μg/mL |  |  |  |  | 241.34 (38.58) | 244.73 (33.03) | 252.02 (42.59) | 252.12 (43.99) |
| CNTF, pg/mL | 14.41 (5.73) | 14.36 (3.98) | 14 (0) | 14 (0) | 14 (0) | 14 (0) | 14 (0) | 14 (0) |
| CRP, μg/mL |  |  |  |  | 5.9 (7.07) | 5.54 (4.89) | 5.37 (6.04) | 5.87 (6.64) |
| Cystatin B, ng/mL |  |  |  |  | 9.6 (5.08) | 10.58 (4.8) | 9.93 (8.57) | 10.22 (5.41) |
| Decorin, ng/mL |  |  |  |  | 1.69 (0.38) | 1.65 (0.36) | 1.83 (0.43) | 1.82 (0.39) |
| E-Cad, ng/mL |  |  |  |  | 4995.13 (1501.89) | 4957.81 (1424.77) | 4627.66 (1687.93) | 4909.55 (2289.94) |
| E-Selectin, ng/mL | 11.75 (5.55) | 11.07 (5.56) | 11.84 (5.66) | 11.09 (4.79) | 9.97 (4.23) | 9.82 (4.65) | 9.96 (3.47) | 9.84 (4.13) |
| EGF, pg/mL |  |  |  |  | 240.71 (151.03) | 261.19 (161.2) | 319.65 (201.99) | 305.42 (209.7) |
| EGFR ng/mL |  |  |  |  | 3.64 (0.65) | 3.68 (0.75) | 3.68 (0.85) | 3.74 (0.83) |
| EN-RAGE, ng/mL |  |  |  |  | 78.81 (75.3) | 95.78 (81.2) | 59.36 (53.05) | 61.18 (49.47) |
| ENA-78, ng/mL | 3.34 (2.8) | 3.65 (3.54) | 3.06 (3.21) | 3.24 (3.19) | 2.52 (1.4) | 2.33 (1.27) | 2.36 (1.73) | 2.6 (2.18) |
| Eotaxin-1, pg/mL | 138.13 (88.56) | 155.4 (95.38) | 145.4 (97.64) | 307.45 (203.32) | 228.46 (144.8) | 254.78 (143.5) | 229.16 (81.9) | 522.3 (188.86) |
| Eotaxin-2, pg/mL | 1043.93 (727.51) | 1083.12 (745.3) | 1015.44 (842.35) | 1440.8 (1074.13) | 1355.81 (991.42) | 1336.63 (956.33) | 1154.14 (576.01) | 1968.86 (1063.23) |
| Eotaxin-3, pg/mL |  |  |  |  | 97.5 (0) | 97.5 (0) | 112.02 (95.24) | 97.5 (0) |
| EPR, pg/mL |  |  |  |  | 19.12 (3.98) | 19.82 (6.12) | 20.08 (8.07) | 18.73 (5.02) |
| Factor VII, ng/mL | 402.43 (125.15) | 425.89 (139.9) | 400.5 (129.93) | 415.16 (135.67) | 434.56 (109.93) | 440 (110.61) | 411.51 (135.08) | 436.82 (148.18) |
| FasL, pg/mL | 20.91 (12.82) | 21 (15.35) | 19.18 (7.38) | 21.31 (14.39) | 18.44 (6.01) | 17.5 (0) | 18.19 (4.5) | 17.5 (0) |
| FAS, ng/mL | 18.98 (14.4) | 20.61 (13.85) | 19.19 (13.36) | 25.55 (66.85) |  |  |  |  |
| FGF-4, pg/mL | 153 (0) | 153 (0) | 153 (0) | 153 (0) | 153 (0) | 153 (0) | 153 (0) | 153 (0) |
| FGF basic, pg/mL |  |  |  |  | 10 (0) | 10 (0) | 9.79 (1.37) | 9.79 (1.37) |
| Fib-1C, μg/mL |  |  |  |  | 21.9 (5.31) | 23.54 (6.77) | 21.63 (5.89) | 23.79 (5.99) |
| Fibrinogen, mg/mL |  |  |  |  | 0.23 (0.73) | 0.11 (0.04) | 0.18 (0.54) | 0.1 (0.04) |
| FRTN, ng/mL | 123.05 (126.04) | 105.5 (116.47) | 117.18 (148.47) | 96.51 (108.08) | 155.17 (130.84) | 161.18 (180.93) | 169.79 (153.71) | 167.98 (177.9) |
| G-CSF, pg/mL | 9.64 (7.63) | 10.87 (7.02) | 9.29 (4.82) | 9.89 (5.01) | 8.15 (4.38) | 7.49 (3.6) | 9 (7.35) | 9.02 (6.06) |
| Gelsolin, μg/mL |  |  |  |  | 53.1 (13.56) | 71.8 (25.87) | 66.07 (23.62) | 81.21 (34.04) |
| GM-CSF, pg/mL | 15.5 (0) | 15.5 (0) | 15.5 (0) | 15.5 (0) | 35.5 (0) | 35.5 (0) | 35.5 (0) | 35.5 (0) |
| Haptoglobin, mg/mL |  |  |  |  | 2.26 (0.91) | 2.3 (0.94) | 2.1 (1.17) | 2.02 (0.86) |
| HB-EGF, pg/mL |  |  |  |  | 63.44 (18.06) | 63.85 (16.5) | 57.63 (17.33) | 62.26 (21.09) |
| HCC-4, ng/mL | 4.37 (2.32) | 4.49 (2.43) | 4.31 (2.22) | 4.34 (2.06) |  |  |  |  |
| HGF, ng/mL | 12.26 (7.48) | 13.39 (9.98) | 12.94 (8.35) | 12.8 (8.52) |  |  |  |  |
| I-309, pg/mL | 307.15 (1977.44) | 605.48 (5339.74) | 545.82 (4780.94) | 351.71 (2644.34) | 397.85 (1530.19) | 416.11 (1618.21) | 688.92 (3194.58) | 741.92 (2858.24) |
| ICAM-1, ng/mL | 106.03 (34.4) | 110.72 (38.22) | 111.99 (40.81) | 114.37 (38.53) | 125.95 (38.53) | 131.85 (37.27) | 129.44 (47.01) | 134.47 (59.57) |
| IFN-γ, pg/mL | 1.81 (0.16) | 1.82 (0.29) | 1.89 (0.85) | 1.92 (1.2) | 1.2 (0.29) | 1.15 (0) | 1.19 (0.28) | 1.29 (0.73) |
| IgA, mg/mL |  |  |  |  | 2.52 (1.23) | 2.63 (1.2) | 2.32 (0.97) | 2.37 (1.05) |
| IgE, Units/mL | 194.23 (317.31) | 196.01 (313.64) | 232.84 (412.34) | 256.72 (444.92) | 138.83 (293.56) | 157.22 (356.02) | 60.47 (103.23) | 47.05 (71.09) |
| IGFBP-2, ng/mL |  |  |  |  | 116.73 (52.62) | 107.63 (50.03) | 105.91 (51.8) | 101.49 (64.14) |
| IgM, mg/mL |  |  |  |  | 1.64 (0.95) | 1.79 (1.06) | 1.63 (1) | 1.74 (1.28) |
| IL-1α, ng/mL | 0 | 0 (0) | 0 | 0 | 0 | 0 (0) | 0 | 0 |
| IL-1β, pg/mL | 3.52 (1.43) | 3.77 (1.66) | 3.6 (1.82) | 3.61 (1.63) | 3.18 (2.17) | 3.17 (1.44) | 3.2 (1.45) | 3.47 (1.68) |
| IL-1ra, pg/mL | 498.36 (153.71) | 596.77 (212.22) | 487 (179.91) | 595.14 (225.22) | 315.29 (82.52) | 329.24 (125.82) | 278.58 (106.46) | 316.63 (90.32) |
| IL-2, pg/mL | 6.11 (1.5) | 6.04 (0.5) | 6.05 (0.71) | 6 (0) | 4.45 (0) | 4.71 (1.65) | 4.85 (1.87) | 4.7 (1.61) |
| IL-2-Rα, pg/mL | 1967.38 (678.92) | 2170.09 (777.04) | 1944.64 (636.37) | 2110.47 (687.65) | 2552.2 (776.45) | 2833.16 (1597.52) | 2562.55 (1162.09) | 2730.69 (1109.48) |
| IL-3, ng/mL | 0 (0) | 0 (0) | 0 (0) | 0 (0) | 0.01 (0) | 0.01 (0) | 0.01 (0) | 0.01 (0) |
| IL-4, pg/mL | 11.1 (1.36) | 11 (0) | 11.43 (5.07) | 12.23 (14.51) | 14 (0) | 14 (0) | 14 (0) | 14 (0) |
| IL-5, pg/mL | 8.5 (0) | 8.5 (0) | 8.5 (0) | 9.35 (5.6) | 5 (0) | 5 (0) | 5 (0) | 5.91 (5.95) |
| IL-6, pg/mL | 2.21 (1.46) | 2.39 (2.56) | 2.61 (4.75) | 2.24 (1.97) | 8.15 (26.75) | 3.6 (0) | 4.83 (5.73) | 4.68 (6.48) |
| IL-6R, ng/mL | 25.54 (7.64) | 26 (7.83) | 25.66 (8.58) | 26.27 (8.08) | 28.45 (7.74) | 28.96 (8.63) | 29.19 (8.32) | 28.93 (7.98) |
| IL-6Rβ, ng/mL |  |  |  |  | 190.95 (40.12) | 202.98 (42.22) | 196.88 (30.47) | 213.44 (43.07) |
| IL-7, pg/mL | 14.06 (4.59) | 13.83 (3.74) | 13.62 (1.66) | 14.63 (12.01) | 6.24 (1.56) | 6.49 (2.23) | 6.88 (2.84) | 7.14 (3.96) |
| IL-8, pg/mL | 10.04 (12.24) | 14.14 (61.09) | 9.76 (9.62) | 9.33 (6.26) | 43.75 (207.55) | 14.19 (12.78) | 14.06 (13.25) | 19.89 (25.29) |
| IL-10, pg/mL | 2.54 (1.96) | 2.92 (2.04) | 2.76 (2.42) | 3.37 (4.45) | 4 (4.48) | 9.29 (33.97) | 6.65 (14.88) | 4.99 (3.9) |
| IL-12p40, ng/mL | 0.4 (0.15) | 0.48 (0.18) | 0.4 (0.14) | 0.46 (0.15) | 0.39 (0.14) | 0.43 (0.14) | 0.38 (0.13) | 0.44 (0.15) |
| IL-12p70, pg/mL | 21.5 (0) | 21.5 (0) | 21.5 (0) | 21.67 (2.37) | 19 (0) | 19 (0) | 19.58 (3.81) | 19 (0) |
| IL-13, pg/mL | 3.4 (0) | 3.4 (0) | 3.53 (1) | 3.49 (0.94) | 3.1 (0) | 3.1 (0) | 3.61 (3.34) | 3.54 (2.88) |
| IL-15, ng/mL | 0.37 (0.05) | 0.4 (0.13) | 0.37 (0.05) | 0.39 (0.1) | 0.24 (0.09) | 0.25 (0.11) | 0.23 (0.11) | 0.25 (0.12) |
| IL-16, pg/mL | 339.23 (144.64) | 391.82 (147.03) | 339.88 (132.49) | 356.86 (126.09) | 461.61 (195.55) | 511.32 (223.65) | 422.09 (157.93) | 405.47 (129.81) |
| IL-17, pg/mL | 1.94 (0.92) | 2.15 (1.2) | 2.01 (0.98) | 2.12 (1.12) | 1.73 (0.82) | 1.7 (0.71) | 1.54 (0.42) | 1.81 (1.05) |
| IL-18, pg/mL | 194.17 (105.57) | 200.72 (114.73) | 182.9 (82.56) | 182.07 (84.82) | 225.74 (116.62) | 234.78 (121.22) | 231.67 (76.44) | 245.42 (89.09) |
| IL-18bp, ng/mL |  |  |  |  | 13.22 (5.2) | 13.55 (5.67) | 14.01 (5.58) | 14.84 (6.52) |
| IL-23, ng/mL | 1.74 (0.64) | 1.9 (0.66) | 1.75 (0.66) | 1.92 (0.68) | 1.05 (0.39) | 1.13 (0.47) | 0.99 (0.43) | 1.06 (0.38) |
| IP-10, pg/mL | 260.91 (187.47) | 276.74 (179.34) | 272.26 (169.12) | 304.87 (200.39) | 246.71 (100.43) | 241.66 (101.86) | 253.21 (179.61) | 261.14 (156.9) |
| ITAC, pg/mL | 45.75 (57.33) | 45.36 (49.76) | 43.44 (35.93) | 40.29 (26.33) | 39.61 (21.98) | 37.18 (21.25) | 53.86 (58.32) | 45.91 (27.68) |
| LAP-TGF-β1, ng/mL | 9.35 (2.89) | 10.2 (3.54) | 8.9 (2.89) | 9.44 (3.23) | 10.11 (2.67) | 10.11 (2.62) | 9.89 (3.03) | 10.23 (2.74) |
| LTF, ng/mL |  |  |  |  | 39.47 (22.4) | 44.56 (25.41) | 51.21 (34.13) | 49.61 (31.02) |
| MCP-1, pg/mL | 370.36 (190.88) | 368.17 (183.75) | 379.75 (187.47) | 355.95 (175.97) | 466.93 (214.09) | 483.1 (240.49) | 482.12 (184.14) | 514.63 (197.66) |
| MCP-2, pg/mL | 37.62 (20.58) | 38.36 (17.27) | 39.52 (28.49) | 42.29 (29.06) | 45.46 (11.19) | 48.15 (12.7) | 49.67 (36) | 48.6 (20.24) |
| MCP-3, pg/mL | 4.05 (0) | 4.05 (0) | 4.05 (0) | 4.05 (0) | 4.05 (0) | 4.05 (0) | 4.05 (0) | 4.05 (0) |
| MCP-4, pg/mL | 3898.88 (1763.6) | 4081.04 (2102.86) | 3780.58 (1669.16) | 4308.77 (2134.05) | 4009.01 (1605.79) | 3874.64 (1401.14) | 3702.42 (1669.55) | 4220.46 (1776.29) |
| MDC, pg/mL | 537.6 (188.84) | 589.2 (219.81) | 550.3 (187.89) | 607.21 (232.22) | 576.37 (219.13) | 551.39 (187.77) | 557.63 (166.25) | 593.44 (159.41) |
| MICA, pg/mL | 55.55 (33.71) | 56.27 (38.45) | 54.44 (35.04) | 62.96 (42.9) | 59.39 (37.21) | 67.67 (40.68) | 57.1 (36.05) | 77.41 (48.64) |
| Midkine, ng/mL |  |  |  |  | 1.47 (0.94) | 1.45 (1.05) | 1.33 (0.85) | 1.44 (1.44) |
| MIF, ng/mL |  |  |  |  | 0.55 (0.34) | 0.41 (0.4) | 0.57 (0.46) | 0.45 (0.53) |
| MIG, pg/mL | 886.61 (1286.02) | 1040.97 (1766.23) | 930.33 (1139.46) | 976.34 (1107.35) | 1255.36 (711.16) | 1472.29 (1298.6) | 1474.08 (1632.13) | 1625.41 (1366.73) |
| MIP-1α, pg/mL | 47.93 (207.31) | 69.81 (403.4) | 24.59 (16.16) | 29.21 (26.14) | 58.63 (208.73) | 33.52 (20.47) | 90.88 (381.9) | 107.76 (456.94) |
| MIP-1β, pg/mL | 309.85 (172.29) | 313.52 (181.46) | 304.69 (142.36) | 304.03 (135.4) | 471.64 (861.72) | 352.34 (161.84) | 395.74 (227.17) | 391.81 (206.01) |
| MIP-3α, pg/mL | 22.6 (18.85) | 22.98 (19.94) | 26.23 (38.85) | 26.02 (32.26) | 23.41 (13.18) | 24.61 (13.76) | 26.33 (19.22) | 32.14 (24.58) |
| MIP-3β, pg/mL | 315.77 (203.22) | 314.26 (247.96) | 349.94 (282.21) | 323.95 (251.69) | 360.34 (117.11) | 413.47 (400.74) | 419.14 (224.82) | 427.26 (234.95) |
| MMP-1, ng/mL | 12.78 (9.66) | 12.69 (9.15) | 13.07 (10.96) | 12.45 (9.87) | 19.86 (17.66) | 19.97 (16.14) | 18.77 (15.54) | 19.94 (17.23) |
| MMP3, ng/mL | 11.72 (12.47) | 13.18 (15.7) | 12.2 (12.4) | 12.16 (13.31) | 18.53 (9.31) | 21.71 (16.44) | 19.6 (14.64) | 20.36 (10.27) |
| MMP7, ng/mL | 4.23 (1.82) | 4.44 (1.88) | 4.16 (1.56) | 4.37 (1.81) | 5.7 (1.61) | 6.08 (1.76) | 5.33 (2) | 7.08 (9.24) |
| MMP-9, ng/mL | 108.59 (76.84) | 116.45 (76.11) | 106.83 (65.84) | 111.33 (81.7) | 139.54 (79.96) | 142.07 (68.29) | 132.56 (80.28) | 130.36 (67.4) |
| MMP-9 (total), ng/mL | 1141.09 (508.12) | 1181.47 (549.61) | 1149.65 (517.89) | 1130.89 (492.87) | 1427.66 (503.53) | 1402.54 (425.38) | 1394.81 (568.29) | 1460.24 (658.1) |
| MMP-10, ng/mL | 1.28 (0.63) | 1.35 (0.78) | 1.29 (0.63) | 1.16 (0.5) | 1.48 (0.75) | 1.46 (0.55) | 1.53 (0.56) | 1.57 (0.63) |
| MPIF-1, ng/mL | 1.1 (0.42) | 1.21 (0.45) | 1.17 (0.59) | 1.27 (0.57) | 1.36 (0.45) | 1.43 (0.56) | 1.57 (0.74) | 1.56 (0.65) |
| MPO, ng/mL |  |  |  |  | 1495.29 (1048.95) | 1365.53 (774.11) | 1583.25 (1140.29) | 1485.62 (1220.14) |
| MSP, ng/mL |  |  |  |  | 228.49 (98.24) | 222.17 (69.99) | 253.47 (106.44) | 255.89 (92.47) |
| Myoglobin, ng/mL | 32.82 (26.48) | 31.79 (20.87) | 29.58 (17.01) | 31.07 (19.57) | 57.71 (32.16) | 61.66 (41.63) | 50.42 (31.55) | 59.77 (44.02 |
| NGAL, ng/mL |  |  |  |  | 379.95 (182.08) | 388.71 (151.56) | 334.12 (149.37) | 362.56 (157.16) |
| NGFβ, ng/mL |  |  |  |  | 0.04 (0) | 0.04 (0) | 0.04 (0) | 0.04 (0) |
| Nr-CAM, ng/mL |  |  |  |  | 0.62 (0.46) | 0.73 (1.38) | 0.7 (0.38) | 0.69 (0.35) |
| OPG-PM | 4.23 (1.29) | 5.06 (1.4) | 4.23 (1.32) | 5 (1.51) | 6.64 (1.66) | 6.89 (1.91) | 6.4 (3.17) | 6.81 (1.96) |
| PAI-1, ng/mL | 210.3 (80.45) | 208.54 (70.55) | 212.51 (72.85) | 206.92 (76.98) | 203.73 (64.21) | 215.32 (51.38) | 180.74 (54.89) | 190.7 (66.21) |
| PARC, ng/mL | 135.86 (63.16) | 132.25 (65.98) | 127.27 (67.24) | 132.03 (70.48) |  |  |  |  |
| PDGF-BB, pg/mL |  |  |  |  | 11937.63 (4466.43) | 13012.41 (5239.16) | 11033.83 (3985.42) | 11520.03 (4936.42) |
| PECAM-1, ng/mL |  |  |  |  | 64.8 (89.81) | 68.24 (101.67) | 55.51 (12.72) | 57.47 (12.87) |
| PGI, ng/mL |  |  |  |  | 132.15 (97.06) | 141.2 (94.81) | 136.79 (81.56) | 157.02 (105.31) |
| PLGF, pg/mL |  |  |  |  | 20.68 (10.13) | 22.49 (12.96) | 20.86 (11.81) | 22.23 (12.38) |
| PSA-f, ng/mL | 0.03 (0.06) | 0.04 (0.07) | 0.04 (0.07) | 0.04 (0.08) | 0.08 (0.11) | 0.09 (0.13) | 0.07 (0.1) | 0.07 (0.08) |
| RAGE, ng/mL |  |  |  |  | 3.13 (2.22) | 2.69 (1.39) | 2.5 (1.57) | 3.1 (3.08) |
| RANTES, ng/mL | 23.41 (13.08) | 25.27 (15.43) | 24.17 (11.35) | 24.54 (12.12) | 23.31 (10.9) | 23.07 (9.15) | 19.6 (9.26) | 22.35 (8.58) |
| S100-B, ng/mL |  |  |  |  | 0.25 (0) | 0.26 (0.06) | 0.26 (0.05) | 0.26 (0.04) |
| SCF, pg/mL | 398.75 (115.75) | 442.45 (121.83) | 392.13 (111.37) | 434.23 (124.79) | 361.11 (114.22) | 380.07 (90.77) | 388.72 (135.56) | 429.68 (182.85) |
| SCFR, ng/mL |  |  |  |  | 8.3 (1.58) | 8.59 (1.81) | 9.29 (2.06) | 9.08 (2.15) |
| SDF-1, pg/mL | 2696.04 (577.52) | 2803.66 (613.06) | 2727.07 (540.97) | 2858.45 (541.51) | 3346.8 (698.13) | 3264.15 (651.59) | 3273.03 (537.09) | 3321.86 (574.8) |
| SOD-1, ng/mL |  |  |  |  | 21.66 (11.4) | 23.93 (16.46) | 24.86 (13.69) | 26.07 (12.66) |
| Sortilin, ng/mL |  |  |  |  | 8.09 (2.85) | 8.73 (2.69) | 8.52 (2.5) | 8.78 (2.71) |
| SP-D, ng/mL |  |  |  |  | 13.19 (6.07) | 13.73 (6.57) | 13.27 (6.53) | 14.7 (6.65) |
| T-cad, ng/mL |  |  |  |  | 5.42 (2.75) | 5.1 (0.94) | 5.53 (2.32) | 5.37 (1.34) |
| TATI, ng/mL |  |  |  |  | 16.38 (7.92) | 18.52 (10.95) | 15.02 (6.22) | 18.04 (15.02) |
| Tetranectin, μg/mL |  |  |  |  | 16.13 (3.86) | 17.63 (3.33) | 16.26 (3.1) | 18.05 (3.4) |
| TFF3, μg/mL |  |  |  |  | 0.14 (0.05) | 0.15 (0.06) | 0.17 (0.14) | 0.17 (0.11) |
| TGFα, pg/mL |  |  |  |  | 4.8 (1.57) | 5.06 (2.12) | 5.53 (3.6) | 4.98 (3.75) |
| TGFβ3, pg/mL | 97.09 (1159.1) | 14.08 (1.15) | 15.28 (10.85) | 14.14 (2.05) | 14 (0) | 14.76 (4.84) | 14.95 (6.25) | 14 (0) |
| Thrombospondin-1, ng/mL |  |  |  |  | 11558.83 (3454.41) | 12693.9 (2715.74) | 11692.14 (3388.49) | 12946.74 (3454.44) |
| TIMP-1, ng/mL | 150.69 (38.39) | 150.16 (37.65) | 147.01 (35.38) | 146.61 (37.77) | 155.24 (40.35) | 160.83 (34.24) | 147.58 (31.15) | 152.79 (30.76) |
| TIMP-2, ng/mL |  |  |  |  | 76.05 (11.91) | 76.88 (11.3) | 74.12 (14.43) | 76.14 (11.71) |
| TN-C, ng/mL |  |  |  |  | 562.27 (233.97) | 580.39 (248.79) | 539.47 (233.64) | 509.35 (177.88) |
| TNFα, pg/mL | 9.94 (6.92) | 10.13 (6.22) | 9.86 (4.73) | 9.95 (6.33) | 10.19 (6.25) | 11.37 (5.43) | 11.26 (9.21) | 11.05 (5.44) |
| TNFβ, pg/mL | 5.41 (2.78) | 5.71 (4.11) | 5.53 (3.23) | 6 (11.07) | 13.5 (0) | 13.5 (0) | 13.5 (0) | 13.5 (0) |
| TNF-R1, pg/mL | 1503.09 (558.93) | 1580.38 (618.06) | 1540.87 (524.3) | 1590.29 (579.86) | 2054.37 (731.85) | 2017.57 (773.56) | 1809.89 (868.02) | 1926.49 (1125.71) |
| TNF-R2, ng/mL | 6.32 (2.11) | 6.63 (2.02) | 6.37 (1.68) | 6.58 (1.8) | 7.21 (3.3) | 7.38 (3.21) | 7.07 (2.48) | 7.93 (3.62) |
| TRAIL-R3, ng/mL | 14.12 (5.7) | 14.01 (5.88) | 14.52 (5.62) | 13.4 (6.21) |  |  |  |  |
| VCAM-1, ng/mL | 475.72 (125.61) | 467.88 (114.25) | 476.93 (113.29) | 464.4 (113.13) | 541.56 (161.91) | 575.46 (217.97) | 526.4 (132.09) | 547.7 (160.06) |
| VDBP, μg/mL |  |  |  |  | 270.95 (100.63) | 285.58 (102.99) | 294.77 (100.11) | 310.42 (107.46) |
| VEGF, pg/mL | 243.64 (119.5) | 255.45 (122.52) | 244.67 (124.5) | 251.65 (139.17) | 234.32 (168.45) | 248.32 (157.11) | 251.07 (158.77) | 267.63 (194.5) |
| YKL-40, ng/mL |  |  |  |  | 55.08 (49.85) | 65.88 (79.51) | 53.74 (41.99) | 67.53 (63.88) |

Data presented are mean (standard deviation) analyte concentrations.

COPD, chronic obstructive pulmonary disease.
