# Supplementary material for: Modulation of blood inflammatory markers by benralizumab in patients with eosinophilic airway diseases
Source: Respir Res. 2019 Jan 18;20:14. doi: 10.1186/s12931-018-0968-8 (PMC6339432; doi:10.1186/s12931-018-0968-8)
Supplement: Supplementary file 5 — Table S2. Post-hoc analyses: effect of baseline blood eosinophil counts on eosinophil gene signatures. (DOCX 24 kb) [file 12931_2018_968_MOESM5_ESM.docx]

**Table S2** *Post-hoc* analyses: effect of baseline blood eosinophil counts on eosinophil gene signatures

|  | **Asthma cohort** | | | **COPD cohort** |
| --- | --- | --- | --- | --- |
|  | **Eosinophil GSVA score** | | | **Eosinophil GSVA score** |
|  | All samples | EOS-high | EOS-low | All samples |
| **Not adjusting for EOS count** | | | | |
| FDR | <0.05 | <0.05 | <0.05 | <0.05 |
| **Adjusting for EOS count** | | | | |
| FDR | <0.05 | <0.05 | <0.05 | <0.05 |

Due to the small sample size, analogous analyses on EOS-high and EOS-low subgroups were not performed in the COPD cohort.

COPD, chronic obstructive pulmonary disease; EOS, eosinophils; FDR, false discovery rate; GSVA, gene set variation analysis.
